# Supplementary material for: Alcohol-Tolerant Workplace Environments Are a Risk Factor for Young Adult Alcohol Misuse on and off the Job in Australia and the United States
Source: Int J Environ Res Public Health. 2023 Sep 7;20(18):6725. doi: 10.3390/ijerph20186725 (PMC10530761; doi:10.3390/ijerph20186725)
Supplement: Supplementary file 1 [file ijerph-20-06725-s001.zip › Oesterle_Supplemental Table S2.pdf]

Supplemental Table S2. Unadjusted odds ratios (95% Confidence Interval) estimated using logistic regression showing the associations between dimensions of the workplace alcohol environment

		Workplace attitude toward alcohol use		
	Alcohol is available at work	Discouraged vs. not acceptable	Tolerated or encouraged vs. not acceptable	Coworkers use alcohol at work
Workplace does not have a written alcohol policy	2.33 (1.72; 3.17)	3.94 (2.63; 5.90)	5.46 (3.57; 8.34)	2.10 (1.54; 2.87)
Alcohol is available at work	-	4.05 (2.97; 5.52)	8.94 (6.11; 13.09)	4.20 (3.32; 5.30)
Workplace attitude toward alcohol use at work				
Tolerated/encouraged vs. not acceptable	-	-	-	3.25 (2.70; 3.91)
Discouraged vs. not acceptable	-	-	-	5.49 (4.00; 7.53)
