# Supplementary material for: Alcohol-Tolerant Workplace Environments Are a Risk Factor for Young Adult Alcohol Misuse on and off the Job in Australia and the United States
Source: Int J Environ Res Public Health. 2023 Sep 7;20(18):6725. doi: 10.3390/ijerph20186725 (PMC10530761; doi:10.3390/ijerph20186725)
Supplement: Supplementary file 1 [file ijerph-20-06725-s001.zip › Oesterle_Supplemental Tables S10 to S16.pdf]

SUPPLEMENTAL TABLES S10 TO S16: DETAILED REGRESSION RESULTS FOR GENERAL HIGH-RISK DRINKING

Tables S10a-c. Estimates from logistic regression of general high-risk drinking in the past year on alcohol availability at work and covariates

**Table S10a. Model 1**	Victoria								Washington							
							95% C.I.for								95% C.I.for	
	B	S.E.	Wald	df	Sig.	Exp(B)	Lower	Upper	B	S.E.	Wald	df	Sig.	Exp(B)	Lower	Upper
**Alcohol available in the workplace (0/1)**	**0.356**	**0.169**	**4.470**	**1**	**0.035**	**1.428**	**1.026**	**1.987**	**0.904**	**0.230**	**15.525**	**1**	**0.000**	**2.470**	**1.575**	**3.874**
Adolescent alcohol use (1-8)	0.163	0.051	10.109	1	0.001	1.177	1.065	1.302	0.241	0.087	7.740	1	0.005	1.273	1.074	1.509
Male (0/1)	0.945	0.174	29.348	1	0.000	2.572	1.827	3.619	0.563	0.208	7.320	1	0.007	1.757	1.168	2.642
White (0/1)	-0.278	0.294	0.897	1	0.344	0.757	0.426	1.346	-0.058	0.250	0.054	1	0.817	0.944	0.578	1.541
4-year college graduate (0/1)	0.162	0.184	0.767	1	0.381	1.175	0.819	1.687	-0.239	0.244	0.955	1	0.328	0.788	0.488	1.271
Currently full-time student (0/1)	-0.199	0.289	0.475	1	0.491	0.819	0.465	1.444	-0.109	0.334	0.107	1	0.744	0.897	0.466	1.725
Married (0/1)	-2.146	0.739	8.434	1	0.004	0.117	0.027	0.498	-0.620	0.311	3.962	1	0.047	0.538	0.292	0.991
Has child or children (0/1)	-0.757	0.344	4.845	1	0.028	0.469	0.239	0.920	-0.818	0.305	7.198	1	0.007	0.441	0.243	0.802
Financial problems (0/1)	0.283	0.206	1.893	1	0.169	1.327	0.887	1.986	0.187	0.222	0.713	1	0.399	1.206	0.781	1.863
Months of full-time employment (1-12)	0.009	0.019	0.202	1	0.653	1.009	0.971	1.048	-0.029	0.023	1.536	1	0.215	0.972	0.929	1.017
High-risk industry (0/1)	0.049	0.178	0.077	1	0.782	1.051	0.741	1.490	0.265	0.213	1.547	1	0.214	1.303	0.859	1.977
Constant	-1.776	0.397	20.039	1	0.000	0.169			-2.102	0.414	25.826	1	0.000	0.122		

**Table S10b. Model 2**	Victoria								Washington							
							95% C.I.for								95% C.I.for	
	B	S.E.	Wald	df	Sig.	Exp(B)	Lower	Upper	B	S.E.	Wald	df	Sig.	Exp(B)	Lower	Upper
**Alcohol available in the workplace**	**0.046**	**0.189**	**0.061**	**1**	**0.806**	**1.048**	**0.724**	**1.517**	**0.370**	**0.265**	**1.954**	**1**	**0.162**	**1.448**	**0.862**	**2.433**
**Alcohol use or impairment at work (0/1)**	**0.817**	**0.203**	**16.167**	**1**	**0.000**	**2.263**	**1.520**	**3.370**	**1.321**	**0.275**	**23.069**	**1**	**0.000**	**3.748**	**2.186**	**6.427**
Adolescent alcohol use (1-8)	0.149	0.052	8.206	1	0.004	1.161	1.048	1.286	0.252	0.089	7.938	1	0.005	1.286	1.080	1.532
Male (0/1)	0.908	0.177	26.459	1	0.000	2.479	1.754	3.504	0.520	0.213	5.977	1	0.014	1.681	1.109	2.550
White (0/1)	-0.254	0.297	0.731	1	0.392	0.776	0.433	1.389	-0.003	0.255	0.000	1	0.990	0.997	0.604	1.645
4-year college graduate (0/1)	0.100	0.187	0.287	1	0.592	1.105	0.766	1.595	-0.332	0.254	1.710	1	0.191	0.718	0.436	1.180
Currently full-time student (0/1)	-0.153	0.292	0.277	1	0.599	0.858	0.484	1.520	-0.119	0.342	0.121	1	0.728	0.888	0.455	1.734
Married (0/1)	-2.055	0.741	7.681	1	0.006	0.128	0.030	0.548	-0.518	0.316	2.682	1	0.101	0.596	0.321	1.107
Has child or children (0/1)	-0.734	0.346	4.487	1	0.034	0.480	0.244	0.947	-0.841	0.311	7.315	1	0.007	0.431	0.235	0.793
Financial problems (0/1)	0.296	0.207	2.034	1	0.154	1.344	0.895	2.019	0.237	0.228	1.079	1	0.299	1.267	0.811	1.981
Months of full-time employment (1-12)	0.002	0.020	0.013	1	0.910	1.002	0.964	1.042	-0.031	0.024	1.727	1	0.189	0.969	0.925	1.015
High-risk industry (0/1)	0.056	0.180	0.098	1	0.755	1.058	0.743	1.506	0.249	0.217	1.318	1	0.251	1.283	0.838	1.965
Constant	-1.763	0.400	19.419	1	0.000	0.171			-2.243	0.428	27.447	1	0.000	0.106		

**Table S10c. Model 3**	Pooled Sample								State x Alcohol Availability Interaction							
							95% C.I.for								95% C.I.for	
	B	S.E.	Wald	df	Sig.	Exp(B)	Lower	Upper	B	S.E.	Wald	df	Sig.	Exp(B)	Lower	Upper
State (1=VIC, 0=WA)	0.279	0.150	3.454	1	0.063	1.322	0.985	1.773								
**Alcohol available in the workplace**	**0.186**	**0.153**	**1.463**	**1**	**0.226**	**1.204**	**0.891**	**1.626**	**-0.562**	**0.282**	**3.978**	**1**	**0.046**	**0.570**	**0.328**	**0.990**
Alcohol use or impairment at work (0/1)	1.009	0.163	38.351	1	0.000	2.744	1.994	3.777								
Adolescent alcohol use (1-8)	0.172	0.045	14.987	1	0.000	1.188	1.089	1.297								
Male (0/1)	0.714	0.134	28.480	1	0.000	2.043	1.571	2.656								
White (0/1)	-0.124	0.189	0.430	1	0.512	0.883	0.610	1.279								
4-year college graduate (0/1)	-0.056	0.147	0.146	1	0.702	0.945	0.708	1.262								
Currently full-time student (0/1)	-0.127	0.220	0.333	1	0.564	0.881	0.573	1.355								
Married (0/1)	-0.870	0.278	9.772	1	0.002	0.419	0.243	0.723								
Has child or children (0/1)	-0.796	0.228	12.226	1	0.000	0.451	0.289	0.705								
Financial problems (0/1)	0.243	0.152	2.563	1	0.109	1.275	0.947	1.716								
Months of full-time employment (1-12)	-0.012	0.015	0.683	1	0.408	0.988	0.959	1.017								
High-risk industry (0/1)	0.174	0.136	1.631	1	0.202	1.190	0.911	1.554								
Constant	-2.129	0.276	59.334	1	0.000	0.119										

Tables S11a-c. Estimates from logistic regression of general high-risk drinking in the past year on workplace alcohol policy and covariates

**Table S11a. Model 1**	Victoria								Washington							
							95% C.I.for								95% C.I.for	
	B	S.E.	Wald	df	Sig.	Exp(B)	Lower	Upper	B	S.E.	Wald	df	Sig.	Exp(B)	Lower	Upper
**Workplace has a written alcohol policy**																
**Yes** (reference)			0.288	2	0.866						9.963	2	0.007			
**No**	**0.122**	**0.237**	**0.267**	**1**	**0.605**	**1.130**	**0.711**	**1.797**	**0.483**	**0.298**	**2.624**	**1**	**0.105**	**1.621**	**0.904**	**2.908**
**Don't know**	**0.054**	**0.196**	**0.076**	**1**	**0.783**	**1.056**	**0.719**	**1.550**	**0.756**	**0.250**	**9.123**	**1**	**0.003**	**2.130**	**1.304**	**3.478**
Adolescent alcohol use (1-8)	0.169	0.051	10.751	1	0.001	1.184	1.070	1.309	0.298	0.085	12.168	1	0.000	1.347	1.140	1.593
Male (0/1)	0.952	0.174	29.915	1	0.000	2.590	1.842	3.643	0.566	0.208	7.411	1	0.006	1.762	1.172	2.649
White (0/1)	-0.227	0.293	0.599	1	0.439	0.797	0.449	1.415	-0.003	0.249	0.000	1	0.991	0.997	0.612	1.625
4-year college graduate (0/1)	0.209	0.184	1.291	1	0.256	1.233	0.859	1.769	-0.183	0.242	0.574	1	0.449	0.833	0.518	1.337
Currently full-time student (0/1)	-0.238	0.288	0.683	1	0.409	0.788	0.448	1.386	-0.156	0.333	0.220	1	0.639	0.856	0.446	1.642
Married (0/1)	-2.102	0.738	8.117	1	0.004	0.122	0.029	0.519	-0.594	0.309	3.691	1	0.055	0.552	0.301	1.012
Has child or children (0/1)	-0.808	0.343	5.563	1	0.018	0.446	0.228	0.872	-0.866	0.305	8.067	1	0.005	0.421	0.232	0.765
Financial problems (0/1)	0.254	0.206	1.526	1	0.217	1.289	0.861	1.930	0.028	0.219	0.016	1	0.900	1.028	0.669	1.580
Months of full-time employment (1-12)	0.010	0.020	0.256	1	0.613	1.010	0.972	1.049	-0.021	0.023	0.833	1	0.361	0.979	0.936	1.024
High-risk industry (0/1)	0.043	0.180	0.058	1	0.810	1.044	0.734	1.486	0.397	0.210	3.586	1	0.058	1.488	0.986	2.245
Constant	-1.717	0.409	17.647	1	0.000	0.180			-2.300	0.425	29.328	1	0.000	0.100		

**Table S11b. Model 2**	Victoria								Washington							
							95% C.I.for								95% C.I.for	
	B	S.E.	Wald	df	Sig.	Exp(B)	Lower	Upper	B	S.E.	Wald	df	Sig.	Exp(B)	Lower	Upper
**Workplace has a written alcohol policy**																
**Yes** (reference)			0.082	2	0.960						6.846	2	0.033			
**No**	**-0.070**	**0.245**	**0.081**	**1**	**0.775**	**0.932**	**0.577**	**1.508**	**0.192**	**0.316**	**0.371**	**1**	**0.543**	**1.212**	**0.653**	**2.250**
**Don't know**	**-0.020**	**0.200**	**0.010**	**1**	**0.922**	**0.981**	**0.663**	**1.451**	**0.682**	**0.261**	**6.846**	**1**	**0.009**	**1.977**	**1.187**	**3.295**
**Alcohol use or impairment at work (0/1)**	**0.842**	**0.187**	**20.164**	**1**	**0.000**	**2.320**	**1.607**	**3.350**	**1.465**	**0.251**	**33.999**	**1**	**0.000**	**4.329**	**2.645**	**7.084**
Adolescent alcohol use (1-8)	0.147	0.053	7.778	1	0.005	1.158	1.045	1.284	0.282	0.089	10.005	1	0.002	1.326	1.113	1.579
Male (0/1)	0.908	0.177	26.445	1	0.000	2.480	1.754	3.505	0.541	0.213	6.417	1	0.011	1.717	1.130	2.609
White (0/1)	-0.249	0.296	0.706	1	0.401	0.780	0.436	1.394	-0.032	0.256	0.015	1	0.902	0.969	0.587	1.600
4-year college graduate (0/1)	0.097	0.188	0.268	1	0.605	1.102	0.762	1.593	-0.364	0.256	2.022	1	0.155	0.695	0.421	1.148
Currently full-time student (0/1)	-0.158	0.292	0.293	1	0.589	0.854	0.482	1.513	-0.120	0.345	0.121	1	0.728	0.887	0.452	1.743
Married (0/1)	-2.055	0.741	7.683	1	0.006	0.128	0.030	0.548	-0.488	0.316	2.391	1	0.122	0.614	0.330	1.140
Has child or children (0/1)	-0.735	0.346	4.513	1	0.034	0.480	0.244	0.945	-0.879	0.312	7.951	1	0.005	0.415	0.226	0.765
Financial problems (0/1)	0.288	0.208	1.908	1	0.167	1.333	0.887	2.005	0.166	0.228	0.529	1	0.467	1.181	0.755	1.847
Months of full-time employment (1-12)	0.002	0.020	0.006	1	0.936	1.002	0.963	1.041	-0.025	0.024	1.058	1	0.304	0.976	0.931	1.023
High-risk industry (0/1)	0.055	0.183	0.092	1	0.762	1.057	0.739	1.512	0.307	0.217	1.997	1	0.158	1.360	0.888	2.082
Constant	-1.721	0.411	17.499	1	0.000	0.179			-2.429	0.439	30.559	1	0.000	0.088		

Table S11c. Model 3	Pooled Sample								State x Alcohol Policy Interaction							
							95% C.I.for								95% C.I.for	
	B	S.E.	Wald	df	Sig.	Exp(B)	Lower	Upper	B	S.E.	Wald	df	Sig.	Exp(B)	Lower	Upper
State (1=VIC, 0=WA)	0.279	0.150	3.474	1	0.062	1.322	0.986	1.772								
**Workplace has a written alcohol policy**																
**Yes** (reference)			2.233	2	0.327						5.232	2	0.073			
**No**	**0.055**	**0.193**	**0.081**	**1**	**0.775**	**1.057**	**0.724**	**1.541**	**-0.411**	**0.388**	**1.124**	**1**	**0.289**	**0.663**	**0.310**	**1.418**
**Don't know**	**0.236**	**0.158**	**2.224**	**1**	**0.136**	**1.266**	**0.929**	**1.725**	**-0.705**	**0.317**	**4.957**	**1**	**0.026**	**0.494**	**0.266**	**0.919**
Alcohol use or impairment at work (0/1)	1.078	0.149	52.040	1	0.000	2.940	2.193	3.940								
Adolescent alcohol use (1-8)	0.180	0.045	16.198	1	0.000	1.197	1.097	1.307								
Male (0/1)	0.715	0.134	28.517	1	0.000	2.045	1.573	2.658								
White (0/1)	-0.123	0.189	0.426	1	0.514	0.884	0.611	1.280								
4-year college graduate (0/1)	-0.048	0.147	0.105	1	0.746	0.953	0.714	1.273								
Currently full-time student (0/1)	-0.148	0.220	0.452	1	0.501	0.863	0.561	1.327								
Married (0/1)	-0.859	0.278	9.559	1	0.002	0.423	0.246	0.730								
Has child or children (0/1)	-0.800	0.227	12.407	1	0.000	0.449	0.288	0.701								
Financial problems (0/1)	0.217	0.152	2.049	1	0.152	1.243	0.923	1.674								
Months of full-time employment (1-12)	-0.010	0.015	0.452	1	0.502	0.990	0.961	1.020								
High-risk industry (0/1)	0.198	0.137	2.075	1	0.150	1.219	0.931	1.595								
Constant	-2.183	0.282	59.706	1	0.000	0.113										

Tables S12a-c. Estimates from logistic regression of general high-risk drinking in the past year on workplace alcohol policy that bans alcohol and covariates

**Table S12a. Model 1**	Victoria								Washington							
							95% C.I.for								95% C.I.for	
	B	S.E.	Wald	df	Sig.	Exp(B)	Lower	Upper	B	S.E.	Wald	df	Sig.	Exp(B)	Lower	Upper
**Alcohol policy completely bans alcohol**																
**Yes** (reference)			0.378	3	0.945						13.146	3	0.004			
**No**	**0.068**	**0.226**	**0.090**	**1**	**0.764**	**1.070**	**0.688**	**1.665**	**0.557**	**0.299**	**3.472**	**1**	**0.062**	**1.745**	**0.972**	**3.133**
**Don't know**	**0.085**	**0.221**	**0.146**	**1**	**0.702**	**1.088**	**0.706**	**1.679**	**0.904**	**0.264**	**11.684**	**1**	**0.001**	**2.469**	**1.471**	**4.146**
**No policy**	**0.151**	**0.256**	**0.350**	**1**	**0.554**	**1.163**	**0.705**	**1.921**	**0.636**	**0.310**	**4.198**	**1**	**0.040**	**1.888**	**1.028**	**3.468**
Adolescent alcohol use (1-8)	0.169	0.051	10.787	1	0.001	1.184	1.071	1.310	0.290	0.086	11.272	1	0.001	1.336	1.128	1.583
Male (0/1)	0.951	0.174	29.845	1	0.000	2.588	1.840	3.640	0.562	0.209	7.243	1	0.007	1.754	1.165	2.641
White (0/1)	-0.230	0.293	0.615	1	0.433	0.795	0.448	1.411	-0.040	0.250	0.026	1	0.871	0.960	0.589	1.567
4-year college graduate (0/1)	0.204	0.185	1.216	1	0.270	1.226	0.853	1.763	-0.196	0.242	0.656	1	0.418	0.822	0.511	1.321
Currently full-time student (0/1)	-0.236	0.288	0.668	1	0.414	0.790	0.449	1.390	-0.115	0.334	0.119	1	0.730	0.891	0.464	1.714
Married (0/1)	-2.100	0.738	8.098	1	0.004	0.122	0.029	0.520	-0.599	0.310	3.742	1	0.053	0.549	0.299	1.008
Has child or children (0/1)	-0.809	0.343	5.570	1	0.018	0.445	0.228	0.872	-0.856	0.305	7.882	1	0.005	0.425	0.234	0.772
Financial problems (0/1)	0.258	0.206	1.567	1	0.211	1.294	0.864	1.939	0.044	0.222	0.040	1	0.841	1.045	0.677	1.615
Months of full-time employment (1-12)	0.010	0.020	0.276	1	0.599	1.010	0.972	1.050	-0.020	0.023	0.726	1	0.394	0.980	0.937	1.026
High-risk industry (0/1)	0.043	0.180	0.056	1	0.812	1.044	0.733	1.485	0.364	0.213	2.940	1	0.086	1.440	0.949	2.184
Constant	-1.746	0.421	17.238	1	0.000	0.174			-2.403	0.433	30.739	1	0.000	0.090		

**Table S12b. Model 2**	Victoria								Washington							
							95% C.I.for								95% C.I.for	
	B	S.E.	Wald	df	Sig.	Exp(B)	Lower	Upper	B	S.E.	Wald	df	Sig.	Exp(B)	Lower	Upper
**Alcohol policy completely bans alcohol**																
**Yes** (reference)			0.538	3	0.910						7.901	3	0.048			
**No**	**-0.158**	**0.235**	**0.455**	**1**	**0.500**	**0.854**	**0.539**	**1.352**	**0.305**	**0.311**	**0.962**	**1**	**0.327**	**1.356**	**0.738**	**2.495**
**Don't know**	**-0.094**	**0.228**	**0.170**	**1**	**0.680**	**0.910**	**0.583**	**1.423**	**0.772**	**0.275**	**7.894**	**1**	**0.005**	**2.164**	**1.263**	**3.707**
**No policy**	**-0.145**	**0.269**	**0.291**	**1**	**0.589**	**0.865**	**0.510**	**1.466**	**0.292**	**0.329**	**0.785**	**1**	**0.376**	**1.339**	**0.702**	**2.552**
**Alcohol use or impairment at work (0/1)**	**0.870**	**0.192**	**20.454**	**1**	**0.000**	**2.387**	**1.637**	**3.481**	**1.427**	**0.254**	**31.524**	**1**	**0.000**	**4.165**	**2.531**	**6.854**
Adolescent alcohol use (1-8)	0.145	0.053	7.600	1	0.006	1.156	1.043	1.282	0.273	0.090	9.265	1	0.002	1.314	1.102	1.567
Male (0/1)	0.908	0.177	26.454	1	0.000	2.480	1.754	3.506	0.534	0.214	6.227	1	0.013	1.706	1.121	2.595
White (0/1)	-0.241	0.297	0.662	1	0.416	0.786	0.439	1.405	-0.059	0.257	0.053	1	0.817	0.942	0.570	1.558
4-year college graduate (0/1)	0.107	0.189	0.320	1	0.571	1.113	0.769	1.610	-0.361	0.256	1.993	1	0.158	0.697	0.422	1.151
Currently full-time student (0/1)	-0.162	0.292	0.308	1	0.579	0.850	0.480	1.507	-0.101	0.345	0.086	1	0.769	0.904	0.460	1.777
Married (0/1)	-2.058	0.742	7.697	1	0.006	0.128	0.030	0.546	-0.503	0.317	2.514	1	0.113	0.605	0.324	1.126
Has child or children (0/1)	-0.732	0.346	4.465	1	0.035	0.481	0.244	0.948	-0.859	0.312	7.603	1	0.006	0.424	0.230	0.780
Financial problems (0/1)	0.279	0.209	1.793	1	0.181	1.322	0.878	1.991	0.160	0.230	0.484	1	0.487	1.174	0.747	1.844
Months of full-time employment (1-12)	0.000	0.020	0.000	1	0.983	1.000	0.962	1.040	-0.025	0.024	1.088	1	0.297	0.975	0.930	1.022
High-risk industry (0/1)	0.056	0.183	0.096	1	0.757	1.058	0.740	1.514	0.301	0.219	1.884	1	0.170	1.352	0.879	2.078
Constant	-1.653	0.423	15.284	1	0.000	0.191			-2.465	0.446	30.568	1	0.000	0.085		

**Table S12c. Model 3**	Pooled Sample								State x Alcohol Policy Interaction							
							95% C.I.for								95% C.I.for	
	B	S.E.	Wald	df	Sig.	Exp(B)	Lower	Upper	B	S.E.	Wald	df	Sig.	Exp(B)	Lower	Upper
State (1=VIC, 0=WA)	0.277	0.151	3.342	1	0.068	1.319	0.980	1.774								
**Alcohol policy completely bans alcohol**																
**Yes** (reference)			2.521	3	0.472						7.945	3	0.047			
**No**	**0.089**	**0.186**	**0.231**	**1**	**0.631**	**1.093**	**0.760**	**1.573**	**-0.631**	**0.375**	**2.830**	**1**	**0.092**	**0.532**	**0.255**	**1.110**
**Don't know**	**0.274**	**0.174**	**2.471**	**1**	**0.116**	**1.315**	**0.935**	**1.851**	-0.917	0.343	7.138	1	0.008	0.400	0.204	0.783
**No policy**	**0.094**	**0.207**	**0.208**	**1**	**0.648**	**1.099**	**0.733**	**1.649**	**-0.620**	**0.409**	**2.297**	**1**	**0.130**	**0.538**	**0.241**	**1.199**
Alcohol use or impairment at work (0/1)	0.179	0.045	15.902	1	0.000	1.196	1.095	1.305								
Adolescent alcohol use (1-8)	0.712	0.134	28.186	1	0.000	2.038	1.567	2.650								
Male (0/1)	-0.133	0.189	0.497	1	0.481	0.875	0.604	1.268								
White (0/1)	-0.050	0.148	0.115	1	0.734	0.951	0.712	1.271								
4-year college graduate (0/1)	-0.143	0.220	0.424	1	0.515	0.867	0.563	1.334								
Currently full-time student (0/1)	-0.857	0.278	9.495	1	0.002	0.424	0.246	0.732								
Married (0/1)	-0.793	0.227	12.199	1	0.000	0.452	0.290	0.706								
Has child or children (0/1)	0.212	0.153	1.923	1	0.166	1.236	0.916	1.666								
Financial problems (0/1)	-0.010	0.015	0.443	1	0.506	0.990	0.961	1.020								
Months of full-time employment (1-12)	0.201	0.138	2.140	1	0.143	1.223	0.934	1.602								
High-risk industry (0/1)	1.066	0.152	48.954	1	0.000	2.905	2.155	3.916								
Constant	-2.202	0.287	58.909	1	0.000	0.111										

Tables S13a-c. Estimates from logistic regression of general high-risk drinking in the past year on alcohol-using co-workers and covariates

**Table S13a. Model 1**	Victoria								Washington							
							95% C.I.for								95% C.I.for	
	B	S.E.	Wald	df	Sig.	Exp(B)	Lower	Upper	B	S.E.	Wald	df	Sig.	Exp(B)	Lower	Upper
**Co-workers drink at work or come to work drunk**	**0.681**	**0.174**	**15.361**	**1**	**0.000**	**1.976**	**1.406**	**2.777**	**0.879**	**0.214**	**16.952**	**1**	**0.000**	**2.410**	**1.585**	**3.662**
Adolescent alcohol use (1-8)	0.161	0.052	9.637	1	0.002	1.174	1.061	1.300	0.243	0.086	7.926	1	0.005	1.274	1.076	1.509
Male (0/1)	0.928	0.176	27.926	1	0.000	2.530	1.793	3.570	0.526	0.208	6.405	1	0.011	1.692	1.126	2.542
White (0/1)	-0.227	0.295	0.591	1	0.442	0.797	0.447	1.421	-0.029	0.250	0.014	1	0.907	0.971	0.594	1.586
4-year college graduate (0/1)	0.129	0.186	0.484	1	0.486	1.138	0.791	1.638	-0.201	0.243	0.680	1	0.410	0.818	0.508	1.318
Currently full-time student (0/1)	-0.212	0.292	0.527	1	0.468	0.809	0.457	1.433	-0.175	0.337	0.271	1	0.602	0.839	0.434	1.623
Married (0/1)	-2.095	0.738	8.054	1	0.005	0.123	0.029	0.523	-0.571	0.312	3.343	1	0.068	0.565	0.306	1.042
Has child or children (0/1)	-0.800	0.345	5.375	1	0.020	0.449	0.229	0.884	-0.809	0.307	6.949	1	0.008	0.445	0.244	0.813
Financial problems (0/1)	0.245	0.207	1.401	1	0.237	1.278	0.852	1.917	0.115	0.220	0.274	1	0.601	1.122	0.728	1.729
Months of full-time employment (1-12)	0.007	0.020	0.140	1	0.708	1.007	0.969	1.047	-0.025	0.023	1.159	1	0.282	0.975	0.932	1.021
High-risk industry (0/1)	-0.007	0.180	0.002	1	0.968	0.993	0.698	1.413	0.308	0.211	2.133	1	0.144	1.361	0.900	2.057
Constant	-1.807	0.399	20.459	1	0.000	0.164			-2.181	0.423	26.555	1	0.000	0.113		

**Table S13b. Model 2**	Victoria								Washington							
							95% C.I.for								95% C.I.for	
	B	S.E.	Wald	df	Sig.	Exp(B)	Lower	Upper	B	S.E.	Wald	df	Sig.	Exp(B)	Lower	Upper
**Co-workers drink at work or come to work drunk**	**0.433**	**0.191**	**5.111**	**1**	**0.024**	**1.541**	**1.059**	**2.243**	**0.379**	**0.248**	**2.348**	**1**	**0.125**	**1.461**	**0.900**	**2.374**
**Alcohol use or impairment at work (0/1)**	**0.654**	**0.201**	**10.532**	**1**	**0.001**	**1.923**	**1.296**	**2.854**	**1.297**	**0.277**	**21.888**	**1**	**0.000**	**3.659**	**2.125**	**6.301**
Adolescent alcohol use (1-8)	0.149	0.052	8.088	1	0.004	1.161	1.047	1.286	0.251	0.089	7.980	1	0.005	1.286	1.080	1.530
Male (0/1)	0.899	0.177	25.745	1	0.000	2.456	1.736	3.476	0.501	0.212	5.600	1	0.018	1.650	1.090	2.498
White (0/1)	-0.245	0.297	0.681	1	0.409	0.782	0.437	1.401	0.003	0.255	0.000	1	0.990	1.003	0.608	1.654
4-year college graduate (0/1)	0.078	0.188	0.173	1	0.678	1.081	0.748	1.562	-0.317	0.253	1.568	1	0.210	0.728	0.444	1.196
Currently full-time student (0/1)	-0.157	0.293	0.287	1	0.592	0.855	0.481	1.518	-0.147	0.343	0.184	1	0.668	0.863	0.441	1.689
Married (0/1)	-2.043	0.741	7.613	1	0.006	0.130	0.030	0.553	-0.495	0.316	2.458	1	0.117	0.609	0.328	1.132
Has child or children (0/1)	-0.745	0.347	4.615	1	0.032	0.475	0.240	0.937	-0.843	0.312	7.322	1	0.007	0.430	0.234	0.793
Financial problems (0/1)	0.273	0.208	1.714	1	0.190	1.314	0.873	1.976	0.208	0.227	0.839	1	0.360	1.231	0.789	1.920
Months of full-time employment (1-12)	0.002	0.020	0.011	1	0.917	1.002	0.964	1.042	-0.030	0.024	1.588	1	0.208	0.970	0.926	1.017
High-risk industry (0/1)	0.020	0.182	0.012	1	0.912	1.020	0.715	1.456	0.267	0.216	1.529	1	0.216	1.306	0.855	1.995
Constant	-1.811	0.401	20.447	1	0.000	0.163			-2.265	0.432	27.536	1	0.000	0.104		

**Table S13c. Model 3**	Pooled Sample								State x Co-workers Drink Interaction							
							95% C.I.for								95% C.I.for	
	B	S.E.	Wald	df	Sig.	Exp(B)	Lower	Upper	B	S.E.	Wald	df	Sig.	Exp(B)	Lower	Upper
State (1=VIC, 0=WA)	0.327	0.149	4.832	1	0.028	1.387	1.036	1.856								
**Co-workers drink at work or come to work drunk**	**0.450**	**0.150**	**9.061**	**1**	**0.003**	**1.569**	**1.170**	**2.103**	**-0.221**	**0.276**	**0.641**	**1**	**0.423**	**0.801**	**0.466**	**1.378**
Alcohol use or impairment at work (0/1)	0.889	0.162	30.148	1	0.000	2.433	1.771	3.341								
Adolescent alcohol use (1-8)	0.170	0.045	14.574	1	0.000	1.186	1.086	1.294								
Male (0/1)	0.709	0.134	27.923	1	0.000	2.031	1.562	2.642								
White (0/1)	-0.124	0.189	0.430	1	0.512	0.883	0.610	1.280								
4-year college graduate (0/1)	-0.068	0.148	0.213	1	0.645	0.934	0.699	1.248								
Currently full-time student (0/1)	-0.143	0.220	0.419	1	0.517	0.867	0.563	1.335								
Married (0/1)	-0.857	0.278	9.487	1	0.002	0.424	0.246	0.732								
Has child or children (0/1)	-0.799	0.228	12.254	1	0.000	0.450	0.288	0.704								
Financial problems (0/1)	0.224	0.152	2.168	1	0.141	1.251	0.929	1.684								
Months of full-time employment (1-12)	-0.012	0.015	0.602	1	0.438	0.988	0.960	1.018								
High-risk industry (0/1)	0.147	0.137	1.161	1	0.281	1.159	0.886	1.515								
Constant	-2.179	0.279	61.155	1	0.000	0.113										

Tables S14a-c. Estimates from logistic regression of general high-risk drinking in the past year on workplace alcohol use attitudes and covariates

**Table S14a. Model 1**	Victoria								Washington							
							95% C.I.for								95% C.I.for	
	B	S.E.	Wald	df	Sig.	Exp(B)	Lower	Upper	B	S.E.	Wald	df	Sig.	Exp(B)	Lower	Upper
**Workplace attitude toward drinking at work**																
**Not acceptable** (reference)			17.485	2	0.000						17.949	2	0.000			
**Discouraged**	**0.217**	**0.237**	**0.839**	**1**	**0.360**	**1.242**	**0.781**	**1.977**	**0.718**	**0.281**	**6.510**	**1**	**0.011**	**2.050**	**1.181**	**3.558**
**Tolerated or encouraged**	**0.962**	**0.230**	**17.477**	**1**	**0.000**	**2.618**	**1.667**	**4.111**	**1.227**	**0.323**	**14.454**	**1**	**0.000**	**3.411**	**1.812**	**6.420**
Adolescent alcohol use (1-8)	0.165	0.052	10.203	1	0.001	1.180	1.066	1.306	0.239	0.087	7.546	1	0.006	1.270	1.071	1.506
Male (0/1)	0.933	0.176	27.988	1	0.000	2.541	1.799	3.591	0.518	0.208	6.216	1	0.013	1.679	1.117	2.523
White (0/1)	-0.188	0.294	0.407	1	0.524	0.829	0.465	1.476	0.016	0.250	0.004	1	0.948	1.017	0.622	1.660
4-year college graduate (0/1)	0.175	0.186	0.882	1	0.348	1.191	0.827	1.714	-0.238	0.245	0.942	1	0.332	0.788	0.487	1.275
Currently full-time student (0/1)	-0.191	0.291	0.428	1	0.513	0.826	0.467	1.463	-0.245	0.337	0.529	1	0.467	0.783	0.405	1.515
Married (0/1)	-2.025	0.738	7.531	1	0.006	0.132	0.031	0.561	-0.530	0.312	2.894	1	0.089	0.588	0.319	1.084
Has child or children (0/1)	-0.754	0.344	4.792	1	0.029	0.471	0.240	0.924	-0.812	0.306	7.027	1	0.008	0.444	0.244	0.809
Financial problems (0/1)	0.227	0.207	1.195	1	0.274	1.255	0.835	1.884	0.110	0.220	0.250	1	0.617	1.116	0.725	1.719
Months of full-time employment (1-12)	0.005	0.020	0.062	1	0.804	1.005	0.967	1.044	-0.028	0.023	1.453	1	0.228	0.972	0.929	1.018
High-risk industry (0/1)	-0.048	0.182	0.070	1	0.792	0.953	0.668	1.361	0.293	0.212	1.912	1	0.167	1.341	0.885	2.033
Constant	-1.793	0.400	20.121	1	0.000	0.166			-2.097	0.417	25.268	1	0.000	0.123		

**Table S14b. Model 2**	Victoria								Washington							
							95% C.I.for								95% C.I.for	
	B	S.E.	Wald	df	Sig.	Exp(B)	Lower	Upper	B	S.E.	Wald	df	Sig.	Exp(B)	Lower	Upper
**Workplace attitude toward drinking at work**																
**Not acceptable** (reference)			8.706	2	0.013						2.870	2	0.238			
**Discouraged**	**0.027**	**0.246**	**0.012**	**1**	**0.913**	**1.027**	**0.634**	**1.665**	**0.338**	**0.302**	**1.256**	**1**	**0.262**	**1.403**	**0.776**	**2.535**
**Tolerated or encouraged**	**0.702**	**0.244**	**8.311**	**1**	**0.004**	**2.019**	**1.252**	**3.254**	**0.560**	**0.368**	**2.315**	**1**	**0.128**	**1.750**	**0.851**	**3.597**
**Alcohol use or impairment at work (0/1)**	**0.683**	**0.197**	**11.986**	**1**	**0.001**	**1.980**	**1.345**	**2.915**	**1.295**	**0.274**	**22.360**	**1**	**0.000**	**3.651**	**2.135**	**6.246**
Adolescent alcohol use (1-8)	0.152	0.052	8.425	1	0.004	1.165	1.051	1.291	0.248	0.090	7.693	1	0.006	1.282	1.076	1.528
Male (0/1)	0.904	0.178	25.814	1	0.000	2.469	1.742	3.499	0.500	0.212	5.576	1	0.018	1.649	1.089	2.498
White (0/1)	-0.214	0.297	0.519	1	0.471	0.807	0.451	1.446	0.017	0.255	0.005	1	0.946	1.017	0.617	1.678
4-year college graduate (0/1)	0.097	0.189	0.262	1	0.609	1.101	0.761	1.594	-0.331	0.254	1.695	1	0.193	0.718	0.437	1.182
Currently full-time student (0/1)	-0.142	0.293	0.235	1	0.628	0.868	0.489	1.540	-0.167	0.342	0.237	1	0.626	0.846	0.433	1.655
Married (0/1)	-1.996	0.740	7.275	1	0.007	0.136	0.032	0.579	-0.482	0.316	2.334	1	0.127	0.617	0.333	1.146
Has child or children (0/1)	-0.716	0.348	4.250	1	0.039	0.488	0.247	0.965	-0.837	0.311	7.235	1	0.007	0.433	0.235	0.797
Financial problems (0/1)	0.260	0.209	1.543	1	0.214	1.296	0.861	1.953	0.205	0.227	0.816	1	0.366	1.228	0.787	1.915
Months of full-time employment (1-12)	0.000	0.020	0.000	1	0.983	1.000	0.961	1.039	-0.030	0.024	1.626	1	0.202	0.970	0.926	1.016
High-risk industry (0/1)	-0.016	0.183	0.007	1	0.931	0.984	0.687	1.410	0.259	0.217	1.424	1	0.233	1.295	0.847	1.980
Constant	-1.801	0.402	20.092	1	0.000	0.165			-2.236	0.430	27.023	1	0.000	0.107		

**Table S14c. Model 3**	Pooled Sample								State by Workplace Attitude Interaction							
							95% C.I.for								95% C.I.for	
	B	S.E.	Wald	df	Sig.	Exp(B)	Lower	Upper	B	S.E.	Wald	df	Sig.	Exp(B)	Lower	Upper
State (1=VIC, 0=WA)	0.285	0.149	3.660	1	0.056	1.330	0.993	1.781								
**Workplace attitude toward drinking at work**																
**Not acceptable** (reference)			11.999	2	0.002						1.923	2	0.382			
**Discouraged**	**0.184**	**0.190**	**0.935**	**1**	**0.333**	**1.202**	**0.828**	**1.743**	**-0.514**	**0.371**	**1.917**	**1**	**0.166**	**0.598**	**0.289**	**1.238**
**Tolerated or encouraged**	**0.690**	**0.199**	**11.987**	**1**	**0.001**	**1.994**	**1.349**	**2.947**	**-0.129**	**0.400**	**0.103**	**1**	**0.748**	**0.879**	**0.401**	**1.927**
Alcohol use or impairment at work (0/1)	0.911	0.159	32.839	1	0.000	2.487	1.821	3.395								
Adolescent alcohol use (1-8)	0.172	0.045	14.805	1	0.000	1.188	1.088	1.296								
Male (0/1)	0.706	0.134	27.615	1	0.000	2.026	1.557	2.636								
White (0/1)	-0.098	0.190	0.267	1	0.605	0.907	0.625	1.315								
4-year college graduate (0/1)	-0.057	0.148	0.148	1	0.700	0.945	0.707	1.262								
Currently full-time student (0/1)	-0.137	0.219	0.387	1	0.534	0.872	0.567	1.341								
Married (0/1)	-0.831	0.278	8.926	1	0.003	0.436	0.252	0.751								
Has child or children (0/1)	-0.791	0.228	11.984	1	0.001	0.454	0.290	0.710								
Financial problems (0/1)	0.218	0.152	2.062	1	0.151	1.244	0.923	1.676								
Months of full-time employment (1-12)	-0.013	0.015	0.788	1	0.375	0.987	0.958	1.016								
High-risk industry (0/1)	0.130	0.138	0.890	1	0.345	1.139	0.869	1.491								
Constant	-2.136	0.278	59.056	1	0.000	0.118										

Tables S15a-b. Estimates from logistic regression of general high-risk drinking in the past year on ALL workplace alcohol environment variables

**Table S15a**	Victoria								Washington							
							95% C.I.for								95% C.I.for	
	B	S.E.	Wald	df	Sig.	Exp(B)	Lower	Upper	B	S.E.	Wald	df	Sig.	Exp(B)	Lower	Upper
**Alcohol available in the workplace**	**-0.071**	**0.195**	**0.132**	**1**	**0.716**	**0.931**	**0.635**	**1.366**	**0.267**	**0.278**	**0.920**	**1**	**0.338**	**1.306**	**0.757**	**2.253**
**Workplace has alcohol policy**																
**Yes** (reference)			0.329	2	0.848						5.555	2	0.062			
**No**	**-0.142**	**0.251**	**0.322**	**1**	**0.570**	**0.867**	**0.530**	**1.419**	**0.046**	**0.335**	**0.019**	**1**	**0.890**	**1.047**	**0.543**	**2.020**
**Don't know**	**-0.048**	**0.203**	**0.055**	**1**	**0.815**	**0.954**	**0.641**	**1.419**	**0.619**	**0.269**	**5.299**	**1**	**0.021**	**1.857**	**1.096**	**3.147**
**Workplace attitude toward drinking at work**																
**Not acceptable** (reference)			6.789	2	0.034						0.507	2	0.776			
**Discouraged**	**-0.032**	**0.256**	**0.016**	**1**	**0.901**	**0.968**	**0.586**	**1.601**	**0.108**	**0.319**	**0.115**	**1**	**0.735**	**1.114**	**0.596**	**2.083**
**Tolerated or encouraged**	**0.628**	**0.258**	**5.942**	**1**	**0.015**	**1.874**	**1.131**	**3.105**	**0.286**	**0.406**	**0.496**	**1**	**0.481**	**1.331**	**0.601**	**2.951**
**Co-workers drink at work or come to work drunk**	**0.337**	**0.202**	**2.765**	**1**	**0.096**	**1.400**	**0.942**	**2.083**	**0.269**	**0.264**	**1.041**	**1**	**0.308**	**1.309**	**0.781**	**2.193**
Alcohol use or impairment at work (0/1)	0.609	0.221	7.569	1	0.006	1.838	1.191	2.835	1.121	0.309	13.152	1	0.000	3.069	1.674	5.625
Adolescent alcohol use (1-8)	0.147	0.053	7.691	1	0.006	1.159	1.044	1.286	0.251	0.091	7.643	1	0.006	1.286	1.076	1.536
Male (0/1)	0.901	0.178	25.517	1	0.000	2.463	1.736	3.494	0.567	0.215	6.929	1	0.008	1.762	1.156	2.688
White (0/1)	-0.207	0.300	0.478	1	0.489	0.813	0.452	1.462	-0.073	0.259	0.078	1	0.779	0.930	0.560	1.545
4-year college graduate (0/1)	0.066	0.191	0.118	1	0.731	1.068	0.735	1.552	-0.396	0.257	2.371	1	0.124	0.673	0.407	1.114
Currently full-time student (0/1)	-0.154	0.295	0.273	1	0.601	0.857	0.481	1.528	-0.135	0.345	0.152	1	0.696	0.874	0.444	1.720
Married (0/1)	-2.011	0.741	7.362	1	0.007	0.134	0.031	0.572	-0.494	0.318	2.411	1	0.120	0.610	0.327	1.138
Has child or children (0/1)	-0.727	0.350	4.315	1	0.038	0.484	0.244	0.960	-0.834	0.313	7.090	1	0.008	0.434	0.235	0.802
Financial problems (0/1)	0.238	0.211	1.279	1	0.258	1.269	0.840	1.918	0.203	0.231	0.771	1	0.380	1.225	0.779	1.925
Months of full-time employment (1-12)	-0.002	0.020	0.007	1	0.935	0.998	0.960	1.039	-0.024	0.024	0.946	1	0.331	0.977	0.932	1.024
High-risk industry (0/1)	-0.038	0.187	0.042	1	0.838	0.963	0.668	1.387	0.264	0.220	1.449	1	0.229	1.302	0.847	2.003
Constant	-1.757	0.417	17.711	1	0.000	0.173			-2.452	0.443	30.577	1	0.000	0.086		

Table S15b	Pooled Sample								State Interactions[a]							
							95% C.I.for								95% C.I.for	
	B	S.E.	Wald	df	Sig.	Exp(B)	Lower	Upper	B	S.E.	Wald	df	Sig.	Exp(B)	Lower	Upper
State (1=VIC, 0=WA)	0.278	0.152	3.331	1	0.068	1.320	0.980	1.779								
**Alcohol available in the workplace**	**0.053**	**0.159**	**0.113**	**1**	**0.737**	**1.055**	**0.773**	**1.440**	**-0.518**	**0.286**	**3.276**	**1**	**0.070**	**0.596**	**0.340**	**1.044**
**Workplace has alcohol policy**																
**Yes** (reference)			1.832	2	0.400						4.242	2	0.120			
**No**	**-0.068**	**0.199**	**0.116**	**1**	**0.733**	**0.934**	**0.633**	**1.379**	**-0.319**	**0.394**	**0.656**	**1**	**0.418**	**0.727**	**0.335**	**1.574**
**Don't know**	**0.188**	**0.161**	**1.374**	**1**	**0.241**	**1.207**	**0.881**	**1.654**	**-0.653**	**0.321**	**4.136**	**1**	**0.042**	**0.520**	**0.277**	**0.977**
**Workplace attitude toward drinking at work**																
**Not acceptable** (reference)			7.209	2	0.027						1.605	2	0.448			
**Discouraged**	**0.071**	**0.198**	**0.128**	**1**	**0.721**	**1.073**	**0.728**	**1.583**	**-0.473**	**0.374**	**1.602**	**1**	**0.206**	**0.623**	**0.300**	**1.296**
**Tolerated or encouraged**	**0.561**	**0.213**	**6.954**	**1**	**0.008**	**1.752**	**1.155**	**2.658**	**-0.113**	**0.404**	**0.078**	**1**	**0.780**	**0.893**	**0.404**	**1.974**
**Co-workers drink at work or come to work drunk**	**0.349**	**0.158**	**4.878**	**1**	**0.027**	**1.418**	**1.040**	**1.933**	**-0.237**	**0.279**	**0.721**	**1**	**0.396**	**0.789**	**0.457**	**1.363**
Alcohol use or impairment at work (0/1)	0.771	0.178	18.792	1	0.000	2.163	1.526	3.065								
Adolescent alcohol use (1-8)	0.171	0.045	14.470	1	0.000	1.187	1.087	1.297								
Male (0/1)	0.714	0.135	28.054	1	0.000	2.043	1.568	2.661								
White (0/1)	-0.129	0.191	0.458	1	0.498	0.879	0.605	1.277								
4-year college graduate (0/1)	-0.086	0.149	0.330	1	0.565	0.918	0.686	1.229								
Currently full-time student (0/1)	-0.148	0.221	0.451	1	0.502	0.862	0.559	1.329								
Married (0/1)	-0.852	0.280	9.286	1	0.002	0.426	0.247	0.738								
Has child or children (0/1)	-0.771	0.229	11.311	1	0.001	0.463	0.295	0.725								
Financial problems (0/1)	0.208	0.153	1.832	1	0.176	1.231	0.911	1.662								
Months of full-time employment (1-12)	-0.011	0.015	0.527	1	0.468	0.989	0.960	1.019								
High-risk industry (0/1)	0.132	0.139	0.899	1	0.343	1.141	0.869	1.498								
Constant	-2.221	0.286	60.380	1	0.000	0.108										

Note: [a] Only one interaction between state and each of the workplace alcohol environment variables was included in the model at a time.

Table S16a-b. Estimates from logistic regression of general high-risk drinking in the past year on ALL workplace alcohol environment variables

Table S16a	Victoria								Washington							
							95% C.I.for								95% C.I.for	
	B	S.E.	Wald	df	Sig.	Exp(B)	Lower	Upper	B	S.E.	Wald	df	Sig.	Exp(B)	Lower	Upper
**Alcohol available in the workplace**	**-0.012**	**0.204**	**0.004**	**1**	**0.952**	**0.988**	**0.663**	**1.473**	**0.223**	**0.288**	**0.599**	**1**	**0.439**	**1.249**	**0.711**	**2.195**
**Alcohol policy completely bans alcohol**																
**Yes** (reference)			1.395	3	0.707						6.213	3	0.102			
**No**	**-0.256**	**0.249**	**1.060**	**1**	**0.303**	**0.774**	**0.475**	**1.261**	**0.241**	**0.321**	**0.565**	**1**	**0.452**	**1.273**	**0.679**	**2.386**
**Don't know**	**-0.172**	**0.235**	**0.531**	**1**	**0.466**	**0.842**	**0.531**	**1.336**	**0.693**	**0.284**	**5.963**	**1**	**0.015**	**1.999**	**1.146**	**3.485**
**No policy**	**-0.269**	**0.279**	**0.924**	**1**	**0.336**	**0.765**	**0.442**	**1.322**	**0.133**	**0.351**	**0.144**	**1**	**0.705**	**1.142**	**0.574**	**2.275**
**Workplace attitude toward drinking at work**																
**Not acceptable** (reference)			7.193	2	0.027						0.541	2	0.763			
**Discouraged**	**-0.018**	**0.257**	**0.005**	**1**	**0.945**	**0.982**	**0.593**	**1.626**	**0.117**	**0.319**	**0.135**	**1**	**0.713**	**1.124**	**0.602**	**2.099**
**Tolerated or encouraged**	**0.655**	**0.259**	**6.379**	**1**	**0.012**	**1.926**	**1.158**	**3.203**	**0.294**	**0.405**	**0.525**	**1**	**0.469**	**1.341**	**0.606**	**2.967**
**Co-workers drink at work or come to work drunk**	**0.334**	**0.203**	**2.717**	**1**	**0.099**	**1.397**	**0.939**	**2.079**	**0.287**	**0.264**	**1.183**	**1**	**0.277**	**1.333**	**0.794**	**2.237**
Alcohol use or impairment at work (0/1)	0.622	0.222	7.858	1	0.005	1.863	1.206	2.878	1.098	0.309	12.605	1	0.000	2.999	1.636	5.500
Adolescent alcohol use (1-8)	0.145	0.053	7.382	1	0.007	1.156	1.041	1.283	0.243	0.092	7.032	1	0.008	1.275	1.065	1.526
Male (0/1)	0.900	0.178	25.435	1	0.000	2.460	1.734	3.490	0.557	0.216	6.665	1	0.010	1.746	1.144	2.666
White (0/1)	-0.202	0.299	0.455	1	0.500	0.817	0.454	1.469	-0.096	0.259	0.136	1	0.713	0.909	0.547	1.511
4-year college graduate (0/1)	0.078	0.191	0.167	1	0.683	1.081	0.743	1.573	-0.389	0.257	2.287	1	0.130	0.678	0.410	1.122
Currently full-time student (0/1)	-0.154	0.295	0.273	1	0.601	0.857	0.481	1.528	-0.122	0.346	0.124	1	0.724	0.885	0.449	1.743
Married (0/1)	-2.021	0.742	7.416	1	0.006	0.133	0.031	0.568	-0.501	0.319	2.456	1	0.117	0.606	0.324	1.134
Has child or children (0/1)	-0.715	0.351	4.148	1	0.042	0.489	0.246	0.973	-0.815	0.313	6.762	1	0.009	0.443	0.239	0.818
Financial problems (0/1)	0.225	0.211	1.136	1	0.287	1.253	0.828	1.896	0.189	0.233	0.661	1	0.416	1.208	0.766	1.905
Months of full-time employment (1-12)	-0.004	0.020	0.031	1	0.860	0.996	0.958	1.037	-0.024	0.024	0.988	1	0.320	0.976	0.931	1.024
High-risk industry (0/1)	-0.038	0.187	0.042	1	0.838	0.962	0.667	1.388	0.266	0.221	1.447	1	0.229	1.305	0.846	2.012
Constant	-1.661	0.427	15.149	1	0.000	0.190			-2.475	0.450	30.232	1	0.000	0.084		

Table S16b	Pooled Sample								State Interactions[a]							
							95% C.I.for								95% C.I.for	
	B	S.E.	Wald	df	Sig.	Exp(B)	Lower	Upper	B	S.E.	Wald	df	Sig.	Exp(B)	Lower	Upper
State (1=VIC, 0=WA)	0.285	0.153	3.464	1	0.063	1.330	0.985	1.797								
Alcohol available in the workplace	0.055	0.165	0.111	1	0.740	1.056	0.765	1.459	-0.528	0.286	3.410	1	0.065	0.590	0.336	1.033
Alcohol policy completely bans alcohol																
Yes (reference)			1.868	3	0.600						7.874	3	0.049			
No	-0.062	0.216	0.082	1	0.774	0.940	0.615	1.436	-0.707	0.376	3.532	1	0.060	0.493	0.236	1.031
Don't know	0.194	0.180	1.168	1	0.280	1.214	0.854	1.727	-0.905	0.347	6.777	1	0.009	0.405	0.205	0.800
No policy	0.007	0.194	0.001	1	0.971	1.007	0.688	1.474	-0.562	0.415	1.840	1	0.175	0.570	0.253	1.284
Workplace attitude toward drinking at work																
Not acceptable (reference)			7.149	2	0.028						1.663	2	0.435			
Discouraged	0.073	0.198	0.134	1	0.714	1.075	0.729	1.586	-0.481	0.374	1.658	1	0.198	0.618	0.297	1.286
Tolerated or encouraged	0.560	0.213	6.901	1	0.009	1.750	1.153	2.657	-0.120	0.405	0.088	1	0.767	0.887	0.401	1.960
Co-workers drink at work or come to work drunk	0.354	0.158	4.998	1	0.025	1.424	1.045	1.942	-0.251	0.279	0.807	1	0.369	0.778	0.450	1.345
Alcohol use or impairment at work (0/1)	0.770	0.178	18.694	1	0.000	2.160	1.524	3.063								
Adolescent alcohol use (1-8)	0.169	0.045	14.051	1	0.000	1.184	1.084	1.294								
Male (0/1)	0.710	0.135	27.637	1	0.000	2.034	1.561	2.650								
White (0/1)	-0.135	0.191	0.496	1	0.481	0.874	0.601	1.271								
4-year college graduate (0/1)	-0.083	0.149	0.313	1	0.576	0.920	0.687	1.232								
Currently full-time student (0/1)	-0.147	0.221	0.441	1	0.507	0.864	0.560	1.332								
Married (0/1)	-0.845	0.280	9.132	1	0.003	0.429	0.248	0.743								
Has child or children (0/1)	-0.762	0.229	11.031	1	0.001	0.467	0.298	0.732								
Financial problems (0/1)	0.196	0.154	1.629	1	0.202	1.217	0.900	1.646								
Months of full-time employment (1-12)	-0.011	0.015	0.570	1	0.450	0.989	0.960	1.019								
High-risk industry (0/1)	0.139	0.139	0.989	1	0.320	1.149	0.874	1.510								
Constant	-2.220	0.290	58.568	1	0.000	0.109										

Note: [a] Only one interaction between state and each of the workplace alcohol environment variables was included in the model at a time.
