# Supplementary material for: Alcohol-Tolerant Workplace Environments Are a Risk Factor for Young Adult Alcohol Misuse on and off the Job in Australia and the United States
Source: Int J Environ Res Public Health. 2023 Sep 7;20(18):6725. doi: 10.3390/ijerph20186725 (PMC10530761; doi:10.3390/ijerph20186725)
Supplement: Supplementary file 1 [file ijerph-20-06725-s001.zip › Oesterle_Supplemental Tables S3 to S9.pdf]

SUPPLEMENTAL TABLES S3 TO S9: DETAILED REGRESSION RESULTS FOR ALCOHOL USE/IMPAIRMENT AT WORK

Tables S3a-c. Estimates from logistic regression of alcohol use or impairment at work in the past year on alcohol availability at work and covariates

Table S3a. Model 1	Victoria								Washington							
							95% C.I.for								95% C.I.for	
	B	S.E.	Wald	df	Sig.	Exp(B)	Lower	Upper	B	S.E.	Wald	df	Sig.	Exp(B)	Lower	Upper
**Alcohol available in the workplace (0/1)**	**1.733**	**0.174**	**98.765**	**1**	**0.000**	**5.659**	**4.021**	**7.965**	**1.223**	**0.200**	**37.313**	**1**	**0.000**	**3.399**	**2.295**	**5.033**
Adolescent alcohol use (1-8)	0.342	0.059	33.467	1	0.000	1.408	1.254	1.581	0.120	0.075	2.573	1	0.109	1.128	0.974	1.306
Male (0/1)	0.756	0.176	18.384	1	0.000	2.131	1.508	3.011	0.133	0.162	0.668	1	0.414	1.142	0.831	1.569
White (0/1)	-0.092	0.294	0.098	1	0.754	0.912	0.513	1.622	-0.015	0.193	0.006	1	0.939	0.985	0.675	1.438
4-year college graduate (0/1)	0.405	0.189	4.600	1	0.032	1.499	1.036	2.171	0.129	0.188	0.470	1	0.493	1.137	0.787	1.644
Currently full-time student (0/1)	-0.085	0.279	0.093	1	0.760	0.918	0.532	1.586	0.069	0.262	0.070	1	0.792	1.072	0.641	1.791
Married (0/1)	-0.634	0.361	3.084	1	0.079	0.531	0.262	1.076	-0.728	0.216	11.404	1	0.001	0.483	0.316	0.737
Has child or children (0/1)	-0.564	0.290	3.782	1	0.052	0.569	0.322	1.004	-0.464	0.211	4.843	1	0.028	0.629	0.416	0.950
Financial problems (0/1)	-0.159	0.208	0.584	1	0.445	0.853	0.567	1.283	0.049	0.176	0.078	1	0.781	1.050	0.744	1.483
Months of full-time employment (1-12)	0.002	0.019	0.013	1	0.910	1.002	0.965	1.040	0.020	0.018	1.214	1	0.271	1.021	0.984	1.058
High-risk industry (0/1)	0.182	0.181	1.011	1	0.315	1.200	0.841	1.713	0.402	0.169	5.682	1	0.017	1.494	1.074	2.079
Constant	-1.776	0.398	19.870	1	0.000	0.169			-0.967	0.324	8.928	1	0.003	0.380		

Table S3b. Model 2	Victoria								Washington							
							95% C.I.for								95% C.I.for	
	B	S.E.	Wald	df	Sig.	Exp(B)	Lower	Upper	B	S.E.	Wald	df	Sig.	Exp(B)	Lower	Upper
**Alcohol available in the workplace**	**1.863**	**0.190**	**95.959**	**1**	**0.000**	**6.445**	**4.439**	**9.357**	**1.122**	**0.214**	**27.423**	**1**	**0.000**	**3.071**	**2.018**	**4.674**
**High-risk drinking Age 25 (0,1,2)[a]**	**2.043**	**0.232**	**77.796**	**1**	**0.000**	**7.715**	**4.899**	**12.147**	**2.092**	**0.263**	**63.028**	**1**	**0.000**	**8.100**	**4.833**	**13.576**
Adolescent alcohol use (1-8)	0.301	0.063	23.139	1	0.000	1.351	1.195	1.528	0.050	0.083	0.354	1	0.552	1.051	0.893	1.237
Male (0/1)	0.436	0.193	5.112	1	0.024	1.546	1.060	2.257	-0.030	0.175	0.029	1	0.865	0.971	0.688	1.369
White (0/1)	0.004	0.329	0.000	1	0.989	1.004	0.527	1.915	-0.021	0.208	0.010	1	0.920	0.979	0.651	1.472
4-year college graduate (0/1)	0.333	0.203	2.690	1	0.101	1.396	0.937	2.079	0.215	0.200	1.150	1	0.284	1.240	0.837	1.835
Currently full-time student (0/1)	0.091	0.303	0.090	1	0.764	1.095	0.605	1.981	0.158	0.280	0.319	1	0.572	1.171	0.677	2.026
Married (0/1)	-0.262	0.364	0.519	1	0.471	0.769	0.377	1.570	-0.671	0.228	8.649	1	0.003	0.511	0.327	0.799
Has child or children (0/1)	-0.322	0.308	1.091	1	0.296	0.725	0.396	1.326	-0.287	0.225	1.629	1	0.202	0.751	0.483	1.166
Financial problems (0/1)	-0.308	0.236	1.706	1	0.192	0.735	0.463	1.167	-0.011	0.191	0.003	1	0.954	0.989	0.681	1.437
Months of full-time employment (1-12)	0.003	0.021	0.029	1	0.866	1.003	0.964	1.045	0.031	0.020	2.377	1	0.123	1.031	0.992	1.073
High-risk industry (0/1)	0.183	0.197	0.860	1	0.354	1.201	0.816	1.768	0.390	0.181	4.671	1	0.031	1.477	1.037	2.105
Constant	-2.222	0.442	25.226	1	0.000	0.108			-1.242	0.353	12.358	1	0.000	0.289		

Note: [a] *1 = low risk, 2 = risky, 3 = hazardous or harmful*

**Table S3c. Model 3**	Pooled Sample								State x Alcohol Availability Interaction							
							95% C.I.for								95% C.I.for	
	B	S.E.	Wald	df	Sig.	Exp(B)	Lower	Upper	B	S.E.	Wald	df	Sig.	Exp(B)	Lower	Upper
State (1=VIC, 0=WA)	-0.299	0.144	4.323	1	0.038	0.741	0.559	0.983								
**Alcohol available in the workplace**	**1.531**	**0.139**	**120.675**	**1**	**0.000**	**4.623**	**3.518**	**6.075**	**0.758**	**0.279**	**7.354**	**1**	**0.007**	**2.133**	**1.234**	**3.689**
High-risk drinking Age 25 (0,1,2)[a]	2.029	0.171	140.315	1	0.000	7.607	5.437	10.641								
Adolescent alcohol use (1-8)	0.208	0.048	19.071	1	0.000	1.231	1.121	1.351								
Male (0/1)	0.202	0.128	2.505	1	0.113	1.224	0.953	1.572								
White (0/1)	-0.009	0.174	0.003	1	0.957	0.991	0.705	1.393								
4-year college graduate (0/1)	0.261	0.140	3.457	1	0.063	1.298	0.986	1.709								
Currently full-time student (0/1)	0.127	0.203	0.393	1	0.531	1.135	0.763	1.689								
Married (0/1)	-0.576	0.193	8.912	1	0.003	0.562	0.385	0.821								
Has child or children (0/1)	-0.317	0.179	3.125	1	0.077	0.728	0.513	1.035								
Financial problems (0/1)	-0.114	0.147	0.602	1	0.438	0.892	0.669	1.190								
Months of full-time employment (1-12)	0.018	0.014	1.734	1	0.188	1.019	0.991	1.047								
High-risk industry (0/1)	0.287	0.131	4.803	1	0.028	1.332	1.031	1.721								
Constant	-1.561	0.260	36.001	1	0.000	0.210										

Note : [a] *1 = low risk, 2 = risky, 3 = hazardous or harmful*

Tables S4a-c. Estimates from logistic regression of alcohol use or impairment at work in the past year on workplace alcohol policy and covariates

**Table S4a. Model 1**	Victoria								Washington							
							95% C.I.for								95% C.I.for	
	B	S.E.	Wald	df	Sig.	Exp(B)	Lower	Upper	B	S.E.	Wald	df	Sig.	Exp(B)	Lower	Upper
**Workplace has a written alcohol policy**																
**Yes** (reference)			12.169	2	0.002						2.696	2	0.260			
**No**	**0.829**	**0.243**	**11.666**	**1**	**0.001**	**2.291**	**1.424**	**3.688**	**0.392**	**0.247**	**2.522**	**1**	**0.112**	**1.480**	**0.912**	**2.401**
**Don't know**	**0.031**	**0.180**	**0.030**	**1**	**0.863**	**1.031**	**0.725**	**1.468**	**0.144**	**0.203**	**0.500**	**1**	**0.479**	**1.155**	**0.775**	**1.721**
Adolescent alcohol use (1-8)	0.324	0.056	34.032	1	0.000	1.383	1.240	1.542	0.168	0.072	5.427	1	0.020	1.183	1.027	1.363
Male (0/1)	0.734	0.164	19.922	1	0.000	2.084	1.509	2.876	0.108	0.158	0.467	1	0.494	1.114	0.817	1.520
White (0/1)	0.099	0.277	0.127	1	0.722	1.104	0.641	1.902	0.064	0.189	0.115	1	0.735	1.066	0.736	1.544
4-year college graduate (0/1)	0.548	0.176	9.647	1	0.002	1.729	1.224	2.444	0.239	0.182	1.724	1	0.189	1.271	0.889	1.816
Currently full-time student (0/1)	-0.173	0.260	0.442	1	0.506	0.841	0.505	1.401	-0.012	0.255	0.002	1	0.962	0.988	0.599	1.629
Married (0/1)	-0.413	0.334	1.526	1	0.217	0.662	0.344	1.274	-0.679	0.209	10.563	1	0.001	0.507	0.337	0.764
Has child or children (0/1)	-0.734	0.275	7.111	1	0.008	0.480	0.280	0.823	-0.493	0.207	5.679	1	0.017	0.611	0.407	0.916
Financial problems (0/1)	-0.226	0.194	1.352	1	0.245	0.798	0.545	1.167	-0.074	0.171	0.188	1	0.664	0.929	0.664	1.298
Months of full-time employment (1-12)	0.013	0.018	0.508	1	0.476	1.013	0.978	1.049	0.020	0.018	1.229	1	0.268	1.020	0.985	1.057
High-risk industry (0/1)	0.111	0.171	0.425	1	0.515	1.118	0.800	1.562	0.535	0.163	10.778	1	0.001	1.707	1.241	2.350
Constant	-1.350	0.383	12.461	1	0.000	0.259			-0.937	0.321	8.505	1	0.004	0.392		

**Table S4b. Model 2**	Victoria								Washington							
							95% C.I.for								95% C.I.for	
	B	S.E.	Wald	df	Sig.	Exp(B)	Lower	Upper	B	S.E.	Wald	df	Sig.	Exp(B)	Lower	Upper
**Workplace has a written alcohol policy**																
**Yes** (reference)			11.680	2	0.003						2.009	2	0.366			
**No**	**0.860**	**0.258**	**11.081**	**1**	**0.001**	**2.364**	**1.425**	**3.924**	**0.323**	**0.267**	**1.469**	**1**	**0.226**	**1.382**	**0.819**	**2.331**
**Don't know**	**0.020**	**0.196**	**0.010**	**1**	**0.919**	**1.020**	**0.695**	**1.497**	**-0.115**	**0.227**	**0.258**	**1**	**0.611**	**0.891**	**0.571**	**1.390**
**High-risk drinking Age 25 (0,1,2)[a]**	**1.931**	**0.219**	**77.887**	**1**	**0.000**	**6.900**	**4.493**	**10.596**	**2.173**	**0.263**	**68**	**1**	**0.000**	**8.785**	**5.247**	**14.708**
Adolescent alcohol use (1-8)	0.284	0.057	24.527	1	0.000	1.328	1.187	1.486	0.089	0.081	1.212	1	0.271	1.093	0.933	1.281
Male (0/1)	0.415	0.178	5.435	1	0.020	1.515	1.068	2.148	-0.066	0.173	0.148	1	0.701	0.936	0.667	1.312
White (0/1)	0.241	0.302	0.637	1	0.425	1.273	0.704	2.301	0.089	0.204	0.190	1	0.663	1.093	0.733	1.628
4-year college graduate (0/1)	0.518	0.188	7.591	1	0.006	1.678	1.161	2.426	0.346	0.196	3.124	1	0.077	1.414	0.963	2.076
Currently full-time student (0/1)	-0.049	0.281	0.031	1	0.861	0.952	0.548	1.652	0.058	0.273	0.045	1	0.832	1.060	0.621	1.808
Married (0/1)	-0.034	0.339	0.010	1	0.920	0.966	0.497	1.878	-0.622	0.223	7.750	1	0.005	0.537	0.347	0.832
Has child or children (0/1)	-0.496	0.291	2.914	1	0.088	0.609	0.344	1.076	-0.304	0.221	1.885	1	0.170	0.738	0.478	1.139
Financial problems (0/1)	-0.373	0.215	3.007	1	0.083	0.689	0.452	1.050	-0.094	0.186	0.258	1	0.612	0.910	0.632	1.310
Months of full-time employment (1-12)	0.015	0.019	0.607	1	0.436	1.015	0.978	1.054	0.030	0.020	2.358	1	0.125	1.031	0.992	1.071
High-risk industry (0/1)	0.118	0.184	0.412	1	0.521	1.125	0.785	1.614	0.494	0.176	7.884	1	0.005	1.639	1.161	2.313
Constant	-1.772	0.419	17.887	1	0.000	0.170			-1.203	0.353	11.625	1	0.001	0.300		

Note: [a] *1 = low risk, 2 = risky, 3 = hazardous or harmful*

**Table S4c. Model 3**	Pooled Sample								State x Alcohol Policy Interaction							
							95% C.I.for								95% C.I.for	
	B	S.E.	Wald	df	Sig.	Exp(B)	Lower	Upper	B	S.E.	Wald	df	Sig.	Exp(B)	Lower	Upper
State (1=VIC, 0=WA)	-0.017	0.135	0.0157	1	0.900	0.983	0.755	1.280								
**Workplace has a written alcohol policy**																
**Yes** (reference)			11.311	2	0.003						1.569	2	0.456			
**No**	**0.584**	**0.182**	**10.316**	**1**	**0.001**	**1.794**	**1.256**	**2.562**	**0.458**	**0.366**	**1.567**	**1**	**0.211**	**1.581**	**0.772**	**3.238**
**Don't know**	**-0.039**	**0.146**	**0.071**	**1**	**0.790**	**0.962**	**0.722**	**1.281**	**0.090**	**0.291**	**0.096**	**1**	**0.757**	**1.095**	**0.618**	**1.938**
High-risk drinking Age 25 (0,1,2)[a]	2.028	0.167	147.611	1	0.000	7.598	5.478	10.539								
Adolescent alcohol use (1-8)	0.219	0.045	23.618	1	0.000	1.245	1.139	1.359								
Male (0/1)	0.167	0.122	1.864	1	0.172	1.182	0.930	1.501								
White (0/1)	0.148	0.167	0.788	1	0.375	1.160	0.836	1.608								
4-year college graduate (0/1)	0.422	0.133	10.024	1	0.002	1.525	1.174	1.980								
Currently full-time student (0/1)	-0.005	0.193	0.001	1	0.978	0.995	0.681	1.452								
Married (0/1)	-0.459	0.185	6.194	1	0.013	0.632	0.440	0.907								
Has child or children (0/1)	-0.390	0.173	5.090	1	0.024	0.677	0.482	0.950								
Financial problems (0/1)	-0.206	0.140	2.177	1	0.140	0.813	0.618	1.070								
Months of full-time employment (1-12)	0.023	0.013	2.952	1	0.086	1.023	0.997	1.051								
High-risk industry (0/1)	0.324	0.125	6.669	1	0.010	1.382	1.081	1.767								
Constant	-1.476	0.255	33.606	1	0.000	0.229										

Note : [a] *1 = low risk, 2 = risky, 3 = hazardous or harmful*

Tables S5a-c. Estimates from logistic regression of alcohol use or impairment at work in the past year on workplace alcohol policy that bans alcohol and covariates

**Table S5a. Model 1**	Victoria								Washington							
							95% C.I.for								95% C.I.for	
	B	S.E.	Wald	df	Sig.	Exp(B)	Lower	Upper	B	S.E.	Wald	df	Sig.	Exp(B)	Lower	Upper
**Alcohol policy completely bans alcohol**																
**Yes** (reference)			31.330	3	0.000						29.664	3	0.000			
**No**	**0.958**	**0.219**	**19.200**	**1**	**0.000**	**2.606**	**1.698**	**3.999**	**1.252**	**0.242**	**26.834**	**1**	**0.000**	**3.497**	**2.178**	**5.617**
**Don't know**	**0.456**	**0.205**	**4.939**	**1**	**0.026**	**1.578**	**1.055**	**2.359**	**0.421**	**0.211**	**3.962**	**1**	**0.047**	**1.523**	**1.006**	**2.306**
**No policy**	**1.247**	**0.262**	**22.642**	**1**	**0.000**	**3.480**	**2.082**	**5.816**	**0.677**	**0.254**	**7.138**	**1**	**0.008**	**1.969**	**1.198**	**3.236**
Adolescent alcohol use (1-8)	0.350	0.057	37.329	1	0.000	1.419	1.268	1.587	0.170	0.074	5.273	1	0.022	1.185	1.025	1.370
Male (0/1)	0.740	0.167	19.705	1	0.000	2.096	1.512	2.906	0.119	0.162	0.541	1	0.462	1.127	0.820	1.548
White (0/1)	0.052	0.283	0.034	1	0.854	1.054	0.605	1.833	0.024	0.193	0.016	1	0.901	1.024	0.702	1.494
4-year college graduate (0/1)	0.503	0.179	7.878	1	0.005	1.654	1.164	2.352	0.219	0.186	1.384	1	0.239	1.244	0.864	1.792
Currently full-time student (0/1)	-0.139	0.264	0.277	1	0.598	0.870	0.519	1.460	0.060	0.259	0.053	1	0.817	1.062	0.639	1.763
Married (0/1)	-0.414	0.337	1.502	1	0.220	0.661	0.341	1.281	-0.721	0.214	11.341	1	0.001	0.486	0.320	0.740
Has child or children (0/1)	-0.786	0.279	7.956	1	0.005	0.456	0.264	0.787	-0.515	0.211	5.953	1	0.015	0.598	0.395	0.904
Financial problems (0/1)	-0.182	0.197	0.859	1	0.354	0.833	0.566	1.226	-0.017	0.175	0.009	1	0.923	0.983	0.698	1.385
Months of full-time employment (1-12)	0.020	0.018	1.188	1	0.276	1.020	0.984	1.057	0.026	0.019	1.990	1	0.158	1.027	0.990	1.065
High-risk industry (0/1)	0.094	0.173	0.295	1	0.587	1.099	0.782	1.543	0.429	0.168	6.562	1	0.010	1.536	1.106	2.133
Constant	-1.830	0.407	20.259	1	0.000	0.160			-1.204	0.334	12.962	1	0.000	0.300		

**Table S5b. Model 2**	Victoria								Washington							
							95% C.I.for								95% C.I.for	
	B	S.E.	Wald	df	Sig.	Exp(B)	Lower	Upper	B	S.E.	Wald	df	Sig.	Exp(B)	Lower	Upper
**Alcohol policy completely bans alcohol**																
**Yes** (reference)			31.845	3	0.000						25.797	3	0.000			
**No**	**1.067**	**0.236**	**20.498**	**1**	**0.000**	**2.907**	**1.832**	**4.614**	**1.236**	**0.253**	**23.823**	**1**	**0.000**	**3.441**	**2.095**	**5.652**
**Don't know**	**0.501**	**0.225**	**4.960**	**1**	**0.026**	**1.651**	**1.062**	**2.565**	**0.159**	**0.235**	**0.458**	**1**	**0.498**	**1.172**	**0.740**	**1.858**
**No policy**	**1.335**	**0.282**	**22.481**	**1**	**0.000**	**3.800**	**2.188**	**6.598**	**0.605**	**0.274**	**4.875**	**1**	**0.027**	**1.831**	**1.070**	**3.132**
**High-risk drinking Age 25 (0,1,2)**[a]	**1.974**	**0.223**	**78.571**	**1**	**0.000**	**7.199**	**4.653**	**11.139**	**2.150**	**0.265**	**65.972**	**1**	**0.000**	**8.583**	**5.109**	**14.418**
Adolescent alcohol use (1-8)	0.311	0.059	27.511	1	0.000	1.365	1.215	1.534	0.091	0.083	1.200	1	0.273	1.095	0.931	1.288
Male (0/1)	0.411	0.182	5.125	1	0.024	1.508	1.057	2.153	-0.047	0.176	0.070	1	0.791	0.954	0.676	1.348
White (0/1)	0.184	0.308	0.354	1	0.552	1.201	0.656	2.199	0.050	0.206	0.060	1	0.807	1.052	0.702	1.576
4-year college graduate (0/1)	0.458	0.192	5.703	1	0.017	1.581	1.086	2.302	0.326	0.199	2.670	1	0.102	1.385	0.937	2.047
Currently full-time student (0/1)	-0.024	0.285	0.007	1	0.933	0.976	0.558	1.708	0.151	0.277	0.299	1	0.585	1.163	0.676	2.001
Married (0/1)	-0.023	0.342	0.005	1	0.946	0.977	0.500	1.910	-0.655	0.229	8.148	1	0.004	0.520	0.331	0.814
Has child or children (0/1)	-0.574	0.295	3.800	1	0.051	0.563	0.316	1.003	-0.325	0.226	2.073	1	0.150	0.723	0.464	1.125
Financial problems (0/1)	-0.318	0.218	2.120	1	0.145	0.728	0.475	1.116	-0.035	0.190	0.034	1	0.854	0.966	0.665	1.402
Months of full-time employment (1-12)	0.022	0.019	1.321	1	0.250	1.023	0.984	1.062	0.037	0.020	3.393	1	0.065	1.038	0.998	1.080
High-risk industry (0/1)	0.095	0.187	0.257	1	0.612	1.099	0.762	1.585	0.379	0.181	4.388	1	0.036	1.460	1.025	2.081
Constant	-2.296	0.445	26.584	1	0.000	0.101			-1.477	0.366	16.284	1	0.000	0.228		

Note: [a] *1 = low risk, 2 = risky, 3 = hazardous or harmful*

**Table S5c. Model 3**	Pooled Sample								State x Alcohol Policy Interaction							
							95% C.I.for								95% C.I.for	
	B	S.E.	Wald	df	Sig.	Exp(B)	Lower	Upper	B	S.E.	Wald	df	Sig.	Exp(B)	Lower	Upper
State (1=VIC, 0=WA)	-0.167	0.139	1.445	1	0.229	0.846	0.645	1.111								
**Alcohol policy completely bans alcohol**																
**Yes** (reference)			53.312	3	0.000						4.523	3	0.210			
**No**	**1.098**	**0.169**	**42.176**	**1**	**0.000**	**2.999**	**2.153**	**4.178**	**0.642**	**0.386**	**2.767**	**1**	**0.096**	**1.899**	**0.892**	**4.045**
**Don't know**	**0.345**	**0.159**	**4.724**	**1**	**0.030**	**1.412**	**1.034**	**1.926**	**0.274**	**0.316**	**0.749**	**1**	**0.387**	**1.315**	**0.707**	**2.444**
**No policy**	**0.954**	**0.191**	**24.860**	**1**	**0.000**	**2.597**	**1.784**	**3.778**	**-0.203**	**0.339**	**0.358**	**1**	**0.550**	**0.816**	**0.420**	**1.587**
High-risk drinking Age 25 (0,1,2)[a]	2.034	0.169	145.281	1	0.000	7.641	5.490	10.635								
Adolescent alcohol use (1-8)	0.236	0.046	26.128	1	0.000	1.266	1.157	1.386								
Male (0/1)	0.182	0.124	2.128	1	0.145	1.199	0.940	1.530								
White (0/1)	0.103	0.170	0.367	1	0.545	1.108	0.795	1.545								
4-year college graduate (0/1)	0.368	0.136	7.338	1	0.007	1.445	1.107	1.885								
Currently full-time student (0/1)	0.054	0.196	0.076	1	0.783	1.055	0.719	1.550								
Married (0/1)	-0.471	0.188	6.266	1	0.012	0.624	0.432	0.903								
Has child or children (0/1)	-0.436	0.176	6.141	1	0.013	0.647	0.458	0.913								
Financial problems (0/1)	-0.154	0.142	1.174	1	0.279	0.857	0.648	1.133								
Months of full-time employment (1-12)	0.031	0.014	5.091	1	0.024	1.031	1.004	1.059								
High-risk industry (0/1)	0.255	0.128	3.985	1	0.046	1.291	1.005	1.658								
Constant	-1.794	0.265	45.814	1	0.000	0.166										

Note : [a] *1 = low risk, 2 = risky, 3 = hazardous or harmful*

Tables S6a-c. Estimates from logistic regression of alcohol use or impairment at work in the past year on alcohol-using co-workers and covariates

**Table S6a. Model 1**	Victoria								Washington							
							95% C.I.for								95% C.I.for	
	B	S.E.	Wald	df	Sig.	Exp(B)	Lower	Upper	B	S.E.	Wald	df	Sig.	Exp(B)	Lower	Upper
**Co-workers drink at work or come to work drunk**	**1.499**	**0.192**	**61.089**	**1**	**0.000**	**4.477**	**3.074**	**6.520**	**1.274**	**0.186**	**47.089**	**1**	**0.000**	**3.576**	**2.485**	**5.145**
Adolescent alcohol use (1-8)	0.317	0.057	31.215	1	0.000	1.373	1.228	1.534	0.119	0.075	2.512	1	0.113	1.126	0.972	1.304
Male (0/1)	0.738	0.171	18.591	1	0.000	2.091	1.495	2.924	0.090	0.164	0.304	1	0.581	1.094	0.794	1.508
White (0/1)	0.143	0.287	0.249	1	0.618	1.154	0.657	2.027	0.006	0.194	0.001	1	0.976	1.006	0.687	1.472
4-year college graduate (0/1)	0.396	0.183	4.681	1	0.030	1.486	1.038	2.128	0.178	0.189	0.892	1	0.345	1.195	0.825	1.731
Currently full-time student (0/1)	-0.163	0.271	0.365	1	0.546	0.849	0.500	1.443	0.001	0.265	0.000	1	0.997	1.001	0.595	1.684
Married (0/1)	-0.480	0.348	1.900	1	0.168	0.619	0.313	1.224	-0.695	0.216	10.307	1	0.001	0.499	0.327	0.763
Has child or children (0/1)	-0.718	0.285	6.368	1	0.012	0.488	0.279	0.852	-0.436	0.213	4.194	1	0.041	0.647	0.426	0.981
Financial problems (0/1)	-0.286	0.202	2.000	1	0.157	0.751	0.505	1.117	-0.027	0.177	0.024	1	0.878	0.973	0.688	1.376
Months of full-time employment (1-12)	0.005	0.018	0.062	1	0.804	1.005	0.969	1.042	0.025	0.019	1.767	1	0.184	1.025	0.988	1.064
High-risk industry (0/1)	0.057	0.177	0.105	1	0.746	1.059	0.749	1.497	0.429	0.169	6.451	1	0.011	1.535	1.103	2.137
Constant	-1.499	0.385	15.158	1	0.000	0.223			-1.070	0.327	10.720	1	0.001	0.343		

**Table S6b. Model 2**	Victoria								Washington							
							95% C.I.for								95% C.I.for	
	B	S.E.	Wald	df	Sig.	Exp(B)	Lower	Upper	B	S.E.	Wald	df	Sig.	Exp(B)	Lower	Upper
**Co-workers drink at work or come to work drunk**	**1.394**	**0.202**	**47.601**	**1**	**0.000**	**4.032**	**2.713**	**5.991**	**1.179**	**0.199**	**35.082**	**1**	**0.000**	**3.251**	**2.201**	**4.803**
**High-risk drinking Age 25 (0,1,2)**[a]	**1.842**	**0.221**	**69.238**	**1**	**0.000**	**6.311**	**4.089**	**9.741**	**2.105**	**0.267**	**62.320**	**1**	**0.000**	**8.205**	**4.865**	**13.835**
Adolescent alcohol use (1-8)	0.269	0.059	21.069	1	0.000	1.309	1.167	1.468	0.055	0.083	0.444	1	0.505	1.057	0.898	1.244
Male (0/1)	0.451	0.184	6.008	1	0.014	1.569	1.095	2.250	-0.063	0.177	0.127	1	0.721	0.939	0.664	1.327
White (0/1)	0.251	0.314	0.637	1	0.425	1.285	0.695	2.377	0.021	0.208	0.011	1	0.918	1.022	0.679	1.537
4-year college graduate (0/1)	0.335	0.194	2.968	1	0.085	1.398	0.955	2.046	0.268	0.201	1.769	1	0.183	1.307	0.881	1.938
Currently full-time student (0/1)	-0.028	0.288	0.010	1	0.921	0.972	0.552	1.710	0.111	0.283	0.154	1	0.694	1.117	0.642	1.944
Married (0/1)	-0.124	0.349	0.126	1	0.723	0.884	0.446	1.752	-0.648	0.229	7.991	1	0.005	0.523	0.334	0.820
Has child or children (0/1)	-0.471	0.296	2.526	1	0.112	0.625	0.350	1.116	-0.254	0.226	1.262	1	0.261	0.775	0.498	1.209
Financial problems (0/1)	-0.428	0.223	3.698	1	0.054	0.652	0.421	1.008	-0.064	0.192	0.112	1	0.738	0.938	0.644	1.365
Months of full-time employment (1-12)	0.010	0.020	0.259	1	0.611	1.010	0.972	1.049	0.038	0.020	3.406	1	0.065	1.038	0.998	1.080
High-risk industry (0/1)	0.037	0.189	0.038	1	0.846	1.037	0.716	1.503	0.401	0.181	4.921	1	0.027	1.494	1.048	2.129
Constant	-1.830	0.418	19.193	1	0.000	0.160			-1.395	0.359	15.069	1	0.000	0.248		

Note: [a] *1 = low risk, 2 = risky, 3 = hazardous or harmful*

Table S6c. Model 3	Pooled Sample								State x Co-workers Drink Interaction							
							95% C.I.for								95% C.I.for	
	B	S.E.	Wald	df	Sig.	Exp(B)	Lower	Upper	B	S.E.	Wald	df	Sig.	Exp(B)	Lower	Upper
State (1=VIC, 0=WA)	0.016	0.138	0.014	1	0.907	1.016	0.776	1.331								
**Co-workers drink at work or come to work drunk**	**1.261**	**0.140**	**81.297**	**1**	**0.000**	**3.527**	**2.682**	**4.639**	**0.205**	**0.279**	**0.541**	**1**	**0.462**	**1.228**	**0.710**	**2.123**
High-risk drinking Age 25 (0,1,2)[a]	1.951	0.169	132.855	1	0.000	7.037	5.050	9.806								
Adolescent alcohol use (1-8)	0.201	0.046	18.738	1	0.000	1.222	1.116	1.338								
Male (0/1)	0.182	0.126	2.088	1	0.149	1.199	0.937	1.534								
White (0/1)	0.108	0.172	0.397	1	0.529	1.114	0.796	1.560								
4-year college graduate (0/1)	0.300	0.138	4.734	1	0.030	1.350	1.030	1.768								
Currently full-time student (0/1)	0.033	0.199	0.028	1	0.868	1.034	0.700	1.527								
Married (0/1)	-0.501	0.190	6.966	1	0.008	0.606	0.418	0.879								
Has child or children (0/1)	-0.349	0.177	3.886	1	0.049	0.705	0.499	0.998								
Financial problems (0/1)	-0.208	0.144	2.083	1	0.149	0.812	0.612	1.077								
Months of full-time employment (1-12)	0.024	0.014	3.137	1	0.077	1.025	0.997	1.053								
High-risk industry (0/1)	0.239	0.129	3.438	1	0.064	1.270	0.986	1.635								
Constant	-1.621	0.258	39.488	1	0.000	0.198										

Note : [a] *1 = low risk, 2 = risky, 3 = hazardous or harmful*

Tables S7a-c. Estimates from logistic regression of alcohol use or impairment at work in the past year on workplace alcohol use attitudes and covariates

**Table S7a. Model 1**	Victoria								Washington							
							95% C.I.for								95% C.I.for	
	B	S.E.	Wald	df	Sig.	Exp(B)	Lower	Upper	B	S.E.	Wald	df	Sig.	Exp(B)	Lower	Upper
**Workplace attitude toward drinking at work**																
**Not acceptable** (reference)			44.320	2	0.000						39.164	2	0.000			
**Discouraged**	**0.966**	**0.243**	**15.818**	**1**	**0.000**	**2.627**	**1.632**	**4.228**	**1.115**	**0.254**	**19.242**	**1**	**0.000**	**3.050**	**1.853**	**5.020**
**Tolerated or encouraged**	**1.709**	**0.296**	**33.420**	**1**	**0.000**	**5.521**	**3.093**	**9.853**	**1.741**	**0.359**	**23.515**	**1**	**0.000**	**5.705**	**2.822**	**11.531**
Adolescent alcohol use (1-8)	0.310	0.056	31.128	1	0.000	1.364	1.223	1.521	0.116	0.075	2.367	1	0.124	1.123	0.969	1.302
Male (0/1)	0.720	0.169	18.191	1	0.000	2.054	1.476	2.859	0.068	0.163	0.174	1	0.677	1.070	0.778	1.473
White (0/1)	0.212	0.285	0.550	1	0.458	1.236	0.706	2.162	0.048	0.194	0.060	1	0.806	1.049	0.718	1.533
4-year college graduate (0/1)	0.496	0.181	7.542	1	0.006	1.643	1.153	2.341	0.158	0.188	0.704	1	0.401	1.171	0.810	1.695
Currently full-time student (0/1)	-0.092	0.266	0.119	1	0.730	0.912	0.541	1.537	-0.103	0.267	0.149	1	0.700	0.902	0.535	1.523
Married (0/1)	-0.388	0.339	1.307	1	0.253	0.679	0.349	1.319	-0.645	0.214	9.105	1	0.003	0.524	0.345	0.798
Has child or children (0/1)	-0.655	0.278	5.548	1	0.019	0.520	0.301	0.896	-0.440	0.212	4.315	1	0.038	0.644	0.426	0.975
Financial problems (0/1)	-0.302	0.201	2.267	1	0.132	0.739	0.499	1.095	-0.050	0.176	0.079	1	0.778	0.952	0.673	1.345
Months of full-time employment (1-12)	0.007	0.018	0.141	1	0.707	1.007	0.972	1.043	0.019	0.019	1.074	1	0.300	1.019	0.983	1.057
High-risk industry (0/1)	0.035	0.176	0.041	1	0.840	1.036	0.734	1.462	0.452	0.168	7.254	1	0.007	1.572	1.131	2.184
Constant	-1.507	0.383	15.498	1	0.000	0.222			-0.940	0.324	8.408	1	0.004	0.391		

**Table S7b. Model 2**	Victoria								Washington							
							95% C.I.for								95% C.I.for	
	B	S.E.	Wald	df	Sig.	Exp(B)	Lower	Upper	B	S.E.	Wald	df	Sig.	Exp(B)	Lower	Upper
**Workplace attitude toward drinking at work**																
**Not acceptable** (reference)			32.382	2	0.000						29.777	2	0.000			
**Discouraged**	**0.975**	**0.256**	**14.517**	**1**	**0.000**	**2.652**	**1.606**	**4.380**	**1.070**	**0.271**	**15.554**	**1**	**0.000**	**2.915**	**1.713**	**4.960**
**Tolerated or encouraged**	**1.455**	**0.313**	**21.657**	**1**	**0.000**	**4.284**	**2.321**	**7.907**	**1.562**	**0.380**	**16.925**	**1**	**0.000**	**4.767**	**2.265**	**10.033**
**High-risk drinking Age 25 (0,1,2)**[a]	**1.855**	**0.222**	**69.933**	**1**	**0.000**	**6.394**	**4.139**	**9.876**	**2.086**	**0.264**	**62.389**	**1**	**0.000**	**8.056**	**4.801**	**13.521**
Adolescent alcohol use (1-8)	0.265	0.057	21.335	1	0.000	1.304	1.165	1.460	0.049	0.084	0.348	1	0.555	1.051	0.892	1.238
Male (0/1)	0.438	0.181	5.852	1	0.016	1.550	1.087	2.210	-0.091	0.176	0.265	1	0.607	0.913	0.647	1.290
White (0/1)	0.277	0.312	0.788	1	0.375	1.319	0.716	2.428	0.039	0.208	0.035	1	0.851	1.040	0.691	1.565
4-year college graduate (0/1)	0.448	0.191	5.517	1	0.019	1.566	1.077	2.276	0.278	0.200	1.919	1	0.166	1.320	0.891	1.955
Currently full-time student (0/1)	0.021	0.284	0.006	1	0.940	1.022	0.585	1.784	-0.018	0.282	0.004	1	0.950	0.982	0.565	1.708
Married (0/1)	-0.039	0.342	0.013	1	0.909	0.962	0.492	1.879	-0.581	0.227	6.529	1	0.011	0.559	0.358	0.873
Has child or children (0/1)	-0.440	0.289	2.315	1	0.128	0.644	0.365	1.135	-0.266	0.225	1.398	1	0.237	0.766	0.493	1.191
Financial problems (0/1)	-0.410	0.219	3.494	1	0.062	0.664	0.432	1.020	-0.086	0.191	0.203	1	0.652	0.917	0.630	1.335
Months of full-time employment (1-12)	0.011	0.019	0.310	1	0.578	1.011	0.973	1.050	0.032	0.020	2.569	1	0.109	1.033	0.993	1.074
High-risk industry (0/1)	0.049	0.188	0.068	1	0.794	1.050	0.727	1.517	0.438	0.180	5.911	1	0.015	1.550	1.089	2.207
Constant	-1.820	0.415	19.259	1	0.000	0.162			-1.265	0.355	12.674	1	0.000	0.282		

Note : [a] *1 = low risk, 2 = risky, 3 = hazardous or harmful*

**Table S7c. Model 3**	Pooled Sample								State by Workplace Attitude Interaction							
							95% C.I.for								95% C.I.for	
	B	S.E.	Wald	df	Sig.	Exp(B)	Lower	Upper	B	S.E.	Wald	df	Sig.	Exp(B)	Lower	Upper
State (1=VIC, 0=WA)	-0.018	0.150	0.014	1	0.906	0.982	0.732	1.319								
**Workplace attitude toward drinking at work**																
**Not acceptable** (reference)			29.223	2	0.000						0.138	2	0.933			
**Discouraged**	**1.043**	**0.268**	**15.191**	**1**	**0.000**	**2.838**	**1.680**	**4.796**	**-0.087**	**0.367**	**0.056**	**1**	**0.813**	**0.917**	**0.446**	**1.884**
**Tolerated or encouraged**	**1.535**	**0.377**	**16.625**	**1**	**0.000**	**4.644**	**2.220**	**9.714**	**-0.151**	**0.486**	**0.096**	**1**	**0.757**	**0.860**	**0.332**	**2.232**
High-risk drinking Age 25 (0,1,2)[a]	1.955	0.169	134.399	1	0.000	7.063	5.076	9.830								
Adolescent alcohol use (1-8)	0.199	0.046	19.010	1	0.000	1.221	1.116	1.335								
Male (0/1)	0.167	0.125	1.799	1	0.180	1.182	0.926	1.509								
White (0/1)	0.129	0.171	0.565	1	0.452	1.137	0.813	1.591								
4-year college graduate (0/1)	0.365	0.136	7.168	1	0.007	1.440	1.103	1.881								
Currently full-time student (0/1)	-0.008	0.197	0.002	1	0.968	0.992	0.674	1.461								
Married (0/1)	-0.428	0.187	5.230	1	0.022	0.652	0.452	0.941								
Has child or children (0/1)	-0.347	0.175	3.932	1	0.047	0.707	0.501	0.996								
Financial problems (0/1)	-0.215	0.143	2.262	1	0.133	0.806	0.609	1.068								
Months of full-time employment (1-12)	0.021	0.014	2.267	1	0.132	1.021	0.994	1.048								
High-risk industry (0/1)	0.262	0.128	4.177	1	0.041	1.300	1.011	1.671								
Constant	-1.527	0.257	35.458	1	0.000	0.217										

Note : [a] *1 = low risk, 2 = risky, 3 = hazardous or harmful*

Tables S8a-b. Estimates from logistic regression of alcohol use or impairment at work in the past year on ALL workplace alcohol environment variables

**Table S8a**	Victoria								Washington							
							95% C.I.for								95% C.I.for	
	B	S.E.	Wald	df	Sig.	Exp(B)	Lower	Upper	B	S.E.	Wald	df	Sig.	Exp(B)	Lower	Upper
**Alcohol available in the workplace**	**1.619**	**0.202**	**64.125**	**1**	**0.000**	**5.049**	**3.397**	**7.505**	**0.701**	**0.239**	**8.622**	**1**	**0.003**	**2.015**	**1.262**	**3.217**
**Workplace has alcohol policy**																
**yes**			6.507	2	0.039						2.069	2	0.355			
**no**	**0.670**	**0.292**	**5.278**	**1**	**0.022**	**1.954**	**1.103**	**3.461**	**-0.162**	**0.307**	**0.278**	**1**	**0.598**	**0.851**	**0.466**	**1.552**
**don't know**	**-0.108**	**0.218**	**0.244**	**1**	**0.621**	**0.898**	**0.585**	**1.377**	**-0.348**	**0.246**	**2.005**	**1**	**0.157**	**0.706**	**0.436**	**1.143**
**Workplace attitude toward drinking at work**																
**Not acceptable** (reference)			2.245	2	0.325						8.978	2	0.011			
**Discouraged**	**0.163**	**0.290**	**0.314**	**1**	**0.576**	**1.176**	**0.666**	**2.078**	**0.709**	**0.302**	**5.536**	**1**	**0.019**	**2.033**	**1.126**	**3.671**
**Tolerated or encouraged**	**0.522**	**0.354**	**2.172**	**1**	**0.141**	**1.685**	**0.842**	**3.371**	**0.946**	**0.419**	**5.109**	**1**	**0.024**	**2.576**	**1.134**	**5.850**
**Co-workers drink at work or come to work drunk**	**0.894**	**0.230**	**15.132**	**1**	**0.000**	**2.444**	**1.558**	**3.835**	**0.811**	**0.219**	**13.700**	**1**	**0.000**	**2.250**	**1.465**	**3.458**
High-risk drinking Age 25 (0,1,2)[a]	2.080	0.247	70.805	1	0.000	8.005	4.931	12.995	2.519	0.302	69.623	1	0.000	12.416	6.871	22.435
Adolescent alcohol use (1-8)	0.321	0.065	24.195	1	0.000	1.378	1.213	1.566	0.006	0.086	0.005	1	0.945	1.006	0.850	1.191
Male (0/1)	0.418	0.199	4.416	1	0.036	1.518	1.029	2.242	-0.098	0.182	0.292	1	0.589	0.907	0.635	1.294
White (0/1)	0.081	0.334	0.058	1	0.809	1.084	0.563	2.088	-0.035	0.216	0.026	1	0.873	0.966	0.633	1.475
4-year college graduate (0/1)	0.304	0.212	2.064	1	0.151	1.356	0.895	2.054	0.184	0.209	0.772	1	0.380	1.202	0.797	1.812
Currently full-time student (0/1)	0.085	0.311	0.075	1	0.785	1.089	0.592	2.002	0.097	0.293	0.111	1	0.740	1.102	0.621	1.957
Married (0/1)	-0.176	0.373	0.221	1	0.638	0.839	0.404	1.744	-0.648	0.235	7.585	1	0.006	0.523	0.330	0.829
Has child or children (0/1)	-0.408	0.317	1.657	1	0.198	0.665	0.358	1.237	-0.224	0.231	0.946	1	0.331	0.799	0.509	1.256
Financial problems (0/1)	-0.365	0.239	2.323	1	0.127	0.694	0.434	1.110	-0.001	0.198	0.000	1	0.994	0.999	0.677	1.473
Months of full-time employment (1-12)	-0.004	0.021	0.044	1	0.834	0.996	0.955	1.038	0.035	0.021	2.818	1	0.093	1.036	0.994	1.080
High-risk industry (0/1)	0.019	0.207	0.008	1	0.927	1.019	0.679	1.529	0.295	0.187	2.494	1	0.114	1.343	0.931	1.936
Constant	-2.429	0.472	26.515	1	0.000	0.088			-1.315	0.374	12.361	1	0.000	0.268		

Note: [a] *1 = low risk, 2 = risky, 3 = hazardous or harmful*

**Table S8b**	Pooled Sample								State Interactions[a]							
							95% C.I.for								95% C.I.for	
	B	S.E.	Wald	df	Sig.	Exp(B)	Lower	Upper	B	S.E.	Wald	df	Sig.	Exp(B)	Lower	Upper
State (1=VIC, 0=WA)	-0.267	0.149	3.204	1	0.073	0.766	0.572	1.026								
**Alcohol available in the workplace**	**1.217**	**0.150**	**66.193**	**1**	**0.000**	**3.375**	**2.518**	**4.525**	**0.911**	**0.291**	**9.837**	**1**	**0.002**	**2.488**	**1.407**	**4.396**
**Workplace has alcohol policy**																
**yes**			3.189	2	0.203						3.246	2	0.197			
**no**	**0.214**	**0.204**	**1.105**	**1**	**0.293**	**1.239**	**0.831**	**1.846**	**0.731**	**0.407**	**3.231**	**1**	**0.072**	**2.078**	**0.936**	**4.612**
**don't know**	**-0.183**	**0.158**	**1.342**	**1**	**0.247**	**0.832**	**0.610**	**1.135**	**0.151**	**0.314**	**0.232**	**1**	**0.630**	**1.163**	**0.629**	**2.151**
**Workplace attitude toward drinking at work**																
**Not acceptable** (reference)			8.056	2	0.018						0.132	2	0.936			
**Discouraged**	**0.411**	**0.203**	**4.090**	**1**	**0.043**	**1.508**	**1.013**	**2.247**	**-0.137**	**0.391**	**0.122**	**1**	**0.726**	**0.872**	**0.406**	**1.875**
**Tolerated or encouraged**	**0.620**	**0.265**	**5.479**	**1**	**0.019**	**1.858**	**1.106**	**3.123**	**0.033**	**0.515**	**0.004**	**1**	**0.949**	**1.033**	**0.377**	**2.837**
**Co-workers drink at work or come to work drunk**	**0.815**	**0.155**	**27.481**	**1**	**0.000**	**2.259**	**1.665**	**3.063**	**0.159**	**0.294**	**0.291**	**1**	**0.590**	**1.172**	**0.658**	**2.086**
High-risk drinking Age 25 (0,1,2)[b]	2.221	0.187	141.729	1	0.000	9.219	6.395	13.289								
Adolescent alcohol use (1-8)	0.198	0.049	16.451	1	0.000	1.219	1.108	1.342								
Male (0/1)	0.163	0.131	1.540	1	0.215	1.177	0.910	1.522								
White (0/1)	0.002	0.179	0.000	1	0.991	1.002	0.706	1.423								
4-year college graduate (0/1)	0.207	0.145	2.033	1	0.154	1.230	0.925	1.636								
Currently full-time student (0/1)	0.121	0.208	0.340	1	0.560	1.129	0.751	1.698								
Married (0/1)	-0.536	0.197	7.403	1	0.007	0.585	0.397	0.861								
Has child or children (0/1)	-0.309	0.183	2.864	1	0.091	0.734	0.513	1.050								
Financial problems (0/1)	-0.132	0.150	0.768	1	0.381	0.877	0.653	1.177								
Months of full-time employment (1-12)	0.017	0.014	1.421	1	0.233	1.017	0.989	1.046								
High-risk industry (0/1)	0.155	0.135	1.318	1	0.251	1.168	0.896	1.522								
Constant	-1.661	0.273	36.907	1	0.000	0.190										

Note: [a] Only one interaction between state and each of the workplace alcohol environment variables was included in the model at a time.

[b]1 = low risk, 2 = risky, 3 = hazardous or harmful

Tables S9a-b. Estimates from logistic regression of alcohol use or impairment at work in the past year on ALL workplace alcohol environment variables

**Table S9a**	Victoria								Washington							
							95% C.I.for								95% C.I.for	
	B	S.E.	Wald	df	Sig.	Exp(B)	Lower	Upper	B	S.E.	Wald	df	Sig.	Exp(B)	Lower	Upper
**Alcohol available in the workplace**	1.574	0.213	54.755	1	0.000	4.824	3.180	7.318	0.493	0.250	3.905	1	0.048	1.638	1.004	2.671
**Alcohol policy completely bans alcohol**																
**Yes** (reference)			7.001	3	0.072						15.338	3	0.002			
**No**	0.185	0.273	0.458	1	0.498	1.203	0.704	2.055	0.995	0.272	13.335	1	0.000	2.704	1.585	4.612
**Don't know**	-0.024	0.251	0.009	1	0.923	0.976	0.597	1.595	-0.121	0.253	0.229	1	0.632	0.886	0.539	1.455
**No policy**	0.751	0.315	5.694	1	0.017	2.118	1.143	3.924	0.106	0.314	0.115	1	0.735	1.112	0.601	2.056
**Workplace attitude toward drinking at work**																
**Not acceptable** (reference)			2.018	2	0.365						8.271	2	0.016			
**Discouraged**	0.153	0.291	0.277	1	0.599	1.165	0.659	2.060	0.688	0.306	5.067	1	0.024	1.990	1.093	3.623
**Tolerated or encouraged**	0.498	0.356	1.958	1	0.162	1.645	0.819	3.305	0.923	0.423	4.752	1	0.029	2.516	1.098	5.766
**Co-workers drink at work or come to work drunk**	0.895	0.230	15.166	1	0.000	2.448	1.560	3.842	0.853	0.223	14.588	1	0.000	2.346	1.514	3.633
High-risk drinking Age 25 (0,1,2)[a]	2.088	0.248	70.970	1	0.000	8.066	4.963	13.109	2.514	0.303	68.786	1	0.000	12.356	6.821	22.383
Adolescent alcohol use (1-8)	0.325	0.066	24.538	1	0.000	1.383	1.217	1.573	0.013	0.088	0.022	1	0.882	1.013	0.853	1.203
Male (0/1)	0.416	0.199	4.370	1	0.037	1.516	1.026	2.238	-0.085	0.184	0.211	1	0.646	0.919	0.641	1.318
White (0/1)	0.069	0.335	0.043	1	0.836	1.072	0.556	2.066	-0.044	0.217	0.041	1	0.840	0.957	0.625	1.465
4-year college graduate (0/1)	0.295	0.212	1.930	1	0.165	1.343	0.886	2.037	0.182	0.212	0.736	1	0.391	1.199	0.792	1.815
Currently full-time student (0/1)	0.086	0.311	0.076	1	0.783	1.089	0.593	2.003	0.163	0.295	0.305	1	0.581	1.177	0.660	2.099
Married (0/1)	-0.166	0.373	0.197	1	0.657	0.847	0.408	1.761	-0.656	0.241	7.432	1	0.006	0.519	0.324	0.832
Has child or children (0/1)	-0.426	0.318	1.792	1	0.181	0.653	0.350	1.218	-0.235	0.234	1.008	1	0.315	0.791	0.500	1.250
Financial problems (0/1)	-0.357	0.240	2.219	1	0.136	0.700	0.437	1.119	0.029	0.200	0.022	1	0.883	1.030	0.696	1.523
Months of full-time employment (1-12)	-0.003	0.021	0.017	1	0.896	0.997	0.956	1.040	0.041	0.021	3.742	1	0.053	1.042	0.999	1.086
High-risk industry (0/1)	0.013	0.207	0.004	1	0.950	1.013	0.675	1.520	0.211	0.191	1.220	1	0.269	1.234	0.850	1.794
Constant	-2.492	0.481	26.846	1	0.000	0.083			-1.536	0.384	15.984	1	0.000	0.215		

Note : [a] *1 = low risk, 2 = risky, 3 = hazardous or harmful*

**Table S9b**	Pooled Sample								State Interactions[a]							
							95% C.I.for								95% C.I.for	
	B	S.E.	Wald	df	Sig.	Exp(B)	Lower	Upper	B	S.E.	Wald	df	Sig.	Exp(B)	Lower	Upper
State (1=VIC, 0=WA)	-0.314	0.150	4.354	1	0.037	0.731	0.544	0.981								
**Alcohol available in the workplace**	**1.097**	**0.156**	**49.637**	**1**	**0.000**	**2.995**	**2.207**	**4.064**	**0.892**	**0.292**	**9.327**	**1**	**0.002**	**2.441**	**1.377**	**4.328**
**Alcohol policy completely bans alcohol**																
**Yes** (reference)			11.830	3	0.008						6.161	3	0.104			
**No**	**0.412**	**0.214**	**3.719**	**1**	**0.054**	**1.510**	**0.993**	**2.296**	**0.685**	**0.420**	**2.663**	**1**	**0.103**	**1.985**	**0.871**	**4.520**
**Don't know**	**0.010**	**0.171**	**0.004**	**1**	**0.953**	**1.010**	**0.722**	**1.413**	**0.129**	**0.335**	**0.148**	**1**	**0.701**	**1.138**	**0.589**	**2.195**
**No policy**	**0.552**	**0.188**	**8.603**	**1**	**0.003**	**1.737**	**1.201**	**2.512**	**-0.481**	**0.362**	**1.766**	**1**	**0.184**	**0.618**	**0.304**	**1.256**
**Workplace attitude toward drinking at work**																
**Not acceptable** (reference)			6.775	2	0.034						0.106	2	0.948			
**Discouraged**	**0.382**	**0.204**	**3.499**	**1**	**0.061**	**1.466**	**0.982**	**2.188**	**-0.126**	**0.392**	**0.103**	**1**	**0.748**	**0.882**	**0.409**	**1.902**
**Tolerated or encouraged**	**0.569**	**0.266**	**4.571**	**1**	**0.033**	**1.767**	**1.049**	**2.977**	**0.013**	**0.516**	**0.001**	**1**	**0.980**	**1.013**	**0.369**	**2.784**
**Co-workers drink at work or come to work drunk**	**0.834**	**0.156**	**28.432**	**1**	**0.000**	**2.303**	**1.695**	**3.130**	**0.135**	**0.296**	**0.210**	**1**	**0.647**	**1.145**	**0.641**	**2.045**
High-risk drinking Age 25 (0,1,2)[b]	2.227	0.187	141.497	1	0.000	9.268	6.422	13.376								
Adolescent alcohol use (1-8)	0.207	0.049	17.676	1	0.000	1.230	1.117	1.355								
Male (0/1)	0.173	0.132	1.716	1	0.190	1.188	0.918	1.538								
White (0/1)	-0.010	0.179	0.003	1	0.956	0.990	0.697	1.407								
4-year college graduate (0/1)	0.188	0.146	1.655	1	0.198	1.207	0.906	1.606								
Currently full-time student (0/1)	0.145	0.209	0.485	1	0.486	1.156	0.768	1.741								
Married (0/1)	-0.520	0.199	6.865	1	0.009	0.594	0.403	0.877								
Has child or children (0/1)	-0.330	0.184	3.217	1	0.073	0.719	0.501	1.031								
Financial problems (0/1)	-0.116	0.151	0.591	1	0.442	0.890	0.663	1.197								
Months of full-time employment (1-12)	0.022	0.015	2.271	1	0.132	1.022	0.993	1.052								
High-risk industry (0/1)	0.118	0.136	0.756	1	0.384	1.126	0.862	1.470								
Constant	-1.809	0.279	41.972	1	0.000	0.164										

Note: [a] Only one interaction between state and each of the workplace alcohol environment variables was included in the model at a time.

[b]1 = low risk, 2 = risky, 3 = hazardous or harmful
