# Supplementary material for: Alcohol-Tolerant Workplace Environments Are a Risk Factor for Young Adult Alcohol Misuse on and off the Job in Australia and the United States
Source: Int J Environ Res Public Health. 2023 Sep 7;20(18):6725. doi: 10.3390/ijerph20186725 (PMC10530761; doi:10.3390/ijerph20186725)
Supplement: Supplementary file 1 [file ijerph-20-06725-s001.zip › Oestserle_Supplemental Table S1.pdf]

Supplemental Table S1. Baseline (Grade 7, 2002) characteristics of the analysis sample and the original sample by state

	Victoria, Australia		Washington, USA	
	Analysis Sample[a]	Original Sample	Analysis Sample[a]	Original Sample
	n = 777	n = 984	n = 751	n = 961
Male, %	48.3	50.8	46.7	49.1
White/Australian, %	91.7	90.6	69.1	64.9
Age, mean	12.9	12.9	13.1	13.1
Parental education[b], mean	2.1	2.0	2.5	2.4
Religious service attendance[c], mean	2.2	2.1	2.7	2.7
Grades in school[d], mean	1.8	1.8	2.2	2.2
Used alcohol past year, %	37.4	39.2	20.5	21.1

Notes:

[a] The analysis sample does not include those surveyed at age 25 but not currently working (n = 89 in Victoria and n = 90 in Washington) and those who did not complete an age 25 survey (n = 118 in Victoria and n = 120 in Washington).

[b] 1 = less than high school, 2 = high school degree or some college, 3 = Bachelor’s degree or higher

[c] 1 = never, 2 = rarely, 3 = 1-2 times a month, 4 = about once a week or more often

[d] 1 = very good, 2 = good, 3 = average, 4 = poor, 5 = very poor
